# Absence of oncomodulin increases susceptibility to noise-induced outer hair cell death and alters mitochondrial morphology

**DOI:** 10.3389/fneur.2024.1435749

**Published:** 2024-10-23

**Authors:** Kaitlin E. Murtha, Weintari D. Sese, Kiah Sleiman, Janith Halpage, Pravallika Padyala, Yang Yang, Aubrey J. Hornak, Dwayne D. Simmons

**Affiliations:** Department of Biology, Baylor University, Waco, TX, United States

**Keywords:** mitochondria, Ca^2+^, Ca^2+^ buffer, cell death, cochlea, outer hair cells, oncomodulin, noise-induced hearing loss

## Abstract

Cochlear outer hair cells (OHCs) play a fundamental role in the hearing sensitivity and frequency selectivity of mammalian hearing and are especially vulnerable to noise-induced damage. The OHCs depend on Ca^2+^ homeostasis, which is a balance between Ca^2+^ influx and extrusion, as well as Ca^2+^ buffering by proteins and organelles. Alterations in OHC Ca^2+^ homeostasis is not only an immediate response to noise, but also associated with impaired auditory function. However, there is little known about the contribution of Ca^2+^ buffering proteins and organelles to the vulnerability of OHCs to noise. In this study, we used a knockout (KO) mouse model where oncomodulin (*Ocm*), the major Ca^2+^ binding protein preferentially expressed in OHCs, is deleted. We show that *Ocm* KO mice were more susceptible to noise induced hearing loss compared to wildtype (WT) mice. Following noise exposure (106 dB SPL, 2 h), *Ocm* KO mice had higher threshold shifts and increased OHC loss and TUNEL staining, compared to age-matched WT mice. Mitochondrial morphology was significantly altered in *Ocm* KO OHCs compared to WT OHCs. Before noise exposure, *Ocm* KO OHCs showed decreased mitochondrial abundance, volume, and branching compared to WT OHCs, as measured by immunocytochemical staining of outer mitochondrial membrane protein, TOM20. Following noise exposure, mitochondrial proteins were barely visible in *Ocm* KO OHCs. Using a mammalian cell culture model of prolonged cytosolic Ca^2+^ overload, we show that OCM has protective effects against changes in mitochondrial morphology and apoptosis. These experiments suggest that disruption of Ca^2+^ buffering leads to an increase in noise vulnerability and mitochondrial-associated changes in OHCs.

## 1 Introduction

Noise-induced hearing loss (NIHL) is a preventable form of sensorineural hearing loss caused by long-term exposure of the auditory system to loud sounds (1–3). Approximately 5% of the global population is affected by NIHL, with about 16% of these cases being attributed to occupational noise exposure (4, 5). One of the hallmarks of NIHL is mechanical damage to the sensory structures of the inner ear responsible for audition, such as loss of cochlear outer hair cells (OHCs) (6, 7). OHCs are unable to regenerate in the adult mammalian cochlea. Therefore, loss of OHCs causes significant hearing deficits (8, 9).

Outer hair cells are responsible for amplifying auditory signals and contribute to the frequency selectivity of the mammalian cochlea (10, 11). In addition, OHCs transmit auditory signals to the inner hair cells (IHCs), which relay this information via a neuronal network to the brain (12, 13). The normal function of OHCs is highly dependent on Ca^2+^ and Ca^2+^ homeostasis. In both OHCs and IHCs, auditory signals are converted to electrical impulses through a mechanotransduction (MET) process (14, 15). This process is highly dependent on the influx of potassium (K^+^) and calcium (Ca^2+^) ions through MET channels located on stereocilia found on the apical surface of hair cells (16–18). In addition to contributing to the MET current, Ca^2+^ regulates other aspects of cochlear physiology, including neurotransmitter release at afferent synapses below hair cells (19–21) and has an important role in fast adaptation as well as maturation and innervation of hair cells (22, 23). Taking into consideration the many roles of Ca^2+^ in cochlear physiology, its levels must be tightly regulated temporally and spatially.

The regulation of Ca^2+^ homeostasis in OHCs is achieved through proteins acting as mobile buffers, channels, or pumps and through organelles (24). Defects in proteins involved in Ca^2+^ homeostasis in the cochlea have been associated with various hearing loss pathologies, further highlighting the importance of Ca^2+^ in maintaining auditory function (22, 25–31). To date, oncomodulin (OCM), a small EF-hand Ca^2+^ binding protein (CaBP) belonging to the parvalbumin family of CaBPs (32–34), is the only mobile Ca^2+^ buffer that is associated with a hearing loss phenotype (25, 31, 35, 36). OCM is preferentially expressed in OHCs and is localized to regions with high Ca^2+^ activity (31, 37–39). Organelles such as mitochondria are also involved in OHC Ca^2+^ homeostasis. Similar to OCM, mitochondria are localized near Ca^2+^ hotspots beneath the stereocilia and the synaptic region (24, 40–42). Mitochondria contribute to OHC function by supplying ATP needed for Ca^2+^ extrusion and release of neurotransmitters. Additionally, mitochondria, via the mitochondrial Ca^2+^ uniporter (MCU), maintain optimum Ca^2+^ levels needed for MET (43, 44). Defects in the ability of the mitochondria to buffer Ca^2+^ have been linked to both inherited and acquired hearing loss (45–48).

Alterations in Ca^2+^ homeostasis is one of the suspected mechanisms underlying OHC loss after noise exposure. Elevated intracellular free Ca^2+^ levels in OHCs have been observed following acoustic trauma (49–52). Moreover, cellular Ca^2+^ overload has been associated with the induction of cell death pathways such as apoptosis and necrosis (53–55). Activation of cell death pathways in OHCs after NIHL has been linked to the inability of mitochondria to maintain cytoplasmic Ca^2+^ homeostasis after acoustic overstimulation (48). Further, evidence linking Ca^2+^ overload to sensory cell death after noise exposure is seen in the reduction of hearing thresholds and hair cell death after treatment with Ca^2+^ channel blockers and Ca^2+^ activated proteases (56–61).

The ability of OHCs to compensate for large increases in Ca^2+^ after acoustic overexposure is likely determined by both mobile Ca^2+^ buffers and organelles acting as Ca^2+^ buffers. However, little is known about how mobile protein buffers and organelles interact to respond to OHC Ca^2+^ overload after noise exposure. Using a knockout (KO) mouse model of *Ocm*, which leads to changes in Ca^2+^ dynamics and early progressive hearing loss (23, 25, 31, 62), we show that the absence of *Ocm* also leads to an increased susceptibility to NIHL and OHC loss. Moreover, in *Ocm* KO OHCs, we observe changes in mitochondria, both in control and noise exposed mice. Our findings provide further insight into the mechanisms mediating noise sensitivity and highlight the relationship between Ca^2+^ regulation by CaBPs and mitochondria in OHC survival.

## 2 Methods

### 2.1 Animals

All experiments were performed in compliance with the National Institutes of Health (NIH) guidelines and were approved by the Institutional Animal Care and Use Committee at Baylor University. Mouse colonies were bred and maintained by the Baylor University Vivarium. *Ocm* WT (*Ocm^+/+^*) and *Ocm* KO (*Ocm^−/−^*) *Atoh1*-GCaMP6s mice (63) were previously generated as described by Yang et al. (23). Ai96(RCL-GCaMP6s) mice (Jax strain #024106) contain a floxed-STOP cassette, which when excited by Cre, endogenously express the fluorescent Ca^2+^ indicator dye, GCaMP6s. Ai96(RCL-GCaMP6s) mice were bred with transgenic mice expressing *Atoh1*-driven Cre (23). Then, *Atoh1*-GCaMP6s mice were crossed with *Ocm* WT and KO mice. At young ages (three to 6 weeks-of-age), GCaMP6s expression does not impact auditory function (64).

For genotyping, DNA was extracted from a tail sample using Extract-N-Amp™ Tissue PCR Kit (Sigma, United States), followed by PCR and agarose gel electrophoresis. The following PCR primers were used for genotyping: *Atoh1*-Cre primer pair forward: 5′-CCGGCAGAGTTTACAGAAGC-3′, reverse: 5′-ATG TTT AGC TGG CCC AAA TG-3′; Cre control primer pair forward: 5’-CTA GGC CAC AGA ATT GAA AGA TCT-3′, reverse: 5′-GTA GGT GGA AAT TCT AGC ATC ATC C-3′; GCaMP6s primer pair forward: 5′-AAG GGA GCT GCA GTG GAG TA-3′, reverse: 5’-CCG AAA ATC TGT GGG AAG TC-3′; *Ocm* LoxP primer pair forward: 5’-CTC CAC ACT TCA CCA AGC AG-3′, reverse: 5′-TTT CAT GTT CAG GGA TCA AGT G-3′; *Ocm* mutant primer reverse: 5′-GCT TGG GGA CCC CCT GTC TTC A-3′. Three-to-six week-old mice (both male and female) were used in this study. GCaMP6s fluorescence was used to outline auditory hair cells for immunocytochemical analysis.

### 2.2 Noise exposure

Awake, unrestrained mice were subject to broadband noise (TDT white-noise generator, filtered with a 500 Hz nominal bandwidth centered at 10 kHz) for 2 h at 106 dB SPL in a custom-built soundproof chamber. Mice were placed singly or in pairs in a wire mesh cage with no food or bedding. Sound was delivered using a JBL2446H compression driver coupled to an exponential horn which was about 70 cm above the wire mesh cages in the top of the reverberant box. Unexposed age-matched mice served as controls.

### 2.3 Functional hearing assessments

Auditory brainstem responses (ABRs) and distortion product otoacoustic emissions (DPOAEs) were used to evaluate the hearing ability of animals before and after noise exposure for *Ocm* WT and *Ocm* KO mice. Mice were anesthetized with an intraperitoneal (i.p.) injection of xylazine (20 mg/kg) and ketamine (100 mg/kg). All measurements were obtained in a soundproof chamber with a temperature of 85°F with an ophthalmic ointment (Optixcare eye lube) applied to prevent drying of the eyes as a result of anesthesia. Acoustic stimuli were delivered using a custom-built acoustic assembly (65). ABR potentials were evoked and recorded as previously described (66). Specifically, two electrostatic earphones (EC-1, Tucker Davis Technologies) were used to generate primary tones. A Knowles miniature microphone (EK-3103) was used to record ear canal sound pressure. Outputs and inputs were processed using a digital I-O board (National Instruments, NI PXIe-1073). For ABR measurements, three needle electrodes were inserted subdermally: (1) an active electrode placed in the scalp at the vertex of the skull, (2) a reference electrode overlying the bulla of the ipsilateral ear and (3) a ground electrode above the tail. ABR potentials were evoked with a 5 ms tone burst (0.5 ms rise-fall with a cos2 onset, delivered at 35/s). Sound levels were raised in 10 dB increments from 10 dB below threshold to 80 dB sound pressure level (SPL). The response was amplified, filtered (100 Hz–3 kHz), and averaged (taken from 512 responses) using a LabVIEW (National Instruments)-driven data acquisition system. ABR measurements were obtained at 8, 16, and 32 kHz with threshold levels defined as the lowest SPL in which synchronous waveforms could be detected. DPOAE measurements were taken as previously described (65, 67) at *f2* frequencies of 5.66, 8.00, 11.32, 16.00, 22.56, 32, and 45.2 kHz with threshold levels for each frequency determined as the lowest SPL at which the distortion product (*2f1-f2*) was consistently above the corresponding noise floor. ABR and DPOAE threshold shifts were calculated by subtracting the baseline threshold levels from threshold levels obtained at 2 days post-noise exposure. DPOAE input–output data was collected using the Eaton-Peabody Laboratories Cochlear Function Test Suite (EPL-CFTS).

### 2.4 Cochlear harvest

Mice were anesthetized (Euthasol, 150 mg/kg, i.p.) and transcardially perfused with 4% (w/v) paraformaldehyde (PFA) [EMS, EM Grade, in 1X phosphate buffered saline (PBS), pH 7.4]. Inner ears were removed from the skull and transferred to a petri dish containing chilled 4% PFA. Excess tissue was removed and an insect pin (FST, size 000) was used to poke a small hole in the bony otic capsule at the most apical point of the cochlea. The stapes was removed, and the cochlea was flushed with 4% PFA through the round and oval window using a gel-loading tip (Fisher Scientific). Cochleae were fixed overnight in 4% PFA at 4°C on a rotator. Following fixation, cochleae were decalcified for 5 days in 0.1 M EDTA with solution exchanged every day until cochleae were determined to be fully decalcified by gently squeezing the vestibular portion. Cochleae were then incubated in 50% glycerol (v/v) (Thermo Scientific Chemicals, for molecular biology) solution, then 99.5% glycerol (v/v) and stored at −80°C until use.

### 2.5 Preparation of cochlear tissue

Single cochleae were embedded in a gelatin-agarose solution [5% (w/v) agarose (VWR), 1.5% (w/v) gelatin (EMS, Type B), molecular biology grade with 0.009% (w/v) saline and 30.76 μM sodium azide (Sigma-Aldrich^®^)]. Mid-modiolar tissue sections (50 μm) were created using a Compresstome^®^ vibrating microtome (Precisionary Instruments, VF-500-0Z). Tissue sections were subject to a sucrose gradient (10% (w/v), 20% (w/v), 30% (w/v)), and stored at −80°C or used immediately. For microdissection of cochlear epithelia, cochleae were microdissected as described in Fang et al. (68). Cochlea were microdissected into six pieces and the pieces corresponding to the apical (~8–12 kHz) and basal (28–48 kHz) regions of the cochlea were used (25). The lateral wall and tectorial membranes were removed, and pieces were kept in PBS (containing 30.76 μM sodium azide) until further processing.

### 2.6 TUNEL assay

The Click-iT™ Plus TUNEL Assay Kit (Invitrogen™, C10619, Alexa Fluor™ 647) was used to quantify apoptotic cells in cochlear epithelia and in HEK293T cells. In both cases, the manufacturers’ protocol was followed. TUNEL positive control samples were incubated with 1 unit of DNase I (Invitrogen™) for 30 min at room temperature. For cochlear epithelia only, samples were additionally stained with antibodies described below. Hoechst™ 33342 was used to visualize nuclei.

### 2.7 Immunocytochemistry

Cochlear mid-modiolar tissue sections and microdissected cochlear epithelium were blocked with 5% (v/v) normal horse serum [NHS, Sigma-Aldrich^®^, in 1X PBS with 0.3% (v/v) Triton™ X-100 (Sigma-Aldrich^®^)] at room temperature on a shaker (80 rpm) for 1 h. Samples were incubated overnight at 37°C with primary antibodies diluted in 1% (v/v) NHS-T. The primary antibodies used in this study are as follows: rbMyo7a (Proteus Biosciences, 25–6790, 1:200), rbTOM20 (Invitrogen™, MA5-34964, 1:200), and msIgG1 COXIV (CST^®^, 11967, 1:100). The following day, the appropriate secondary antibodies (diluted in 1% NHS-T) and Alexa Fluor™ Phalloidin 488 (Invitrogen™, A12379, 1:1000) were added and samples were incubated for 2 h at 37°*C. Prior* to the last wash, Hoechst 33342™ (Invitrogen™, H3570, 1:7500) was incubated with the samples for 5 min at room temperature. Cochlear mid-modiolar tissue sections and microdissected pieces were mounted onto SuperFrost^®^ Plus Slides (Fisher Scientific) with VECTASHIELD^®^ mounting media or VECTASHIELD^®^ mounting media with DAPI (Vector Laboratories©) and sealed with clear nail polish. HEK293T cells were mounted onto glass slides with ProLong™ Gold Antifade mountant (Invitrogen™) and left to cure overnight before imaging.

### 2.8 Cell culture maintenance

Human embryonic kidney 293T (HEK293T) cells were cultured in Dulbecco’s modified Eagle’s medium (Gibco™ DMEM, high glucose, pyruvate) supplemented with 10% fetal bovine serum (R&D Systems) and GlutaMAX™ (Gibco™, working concentration of 200 mM l-alanyl-l-glutamine dipeptide). Cells were maintained in 100 mm cell culture dishes (Thermo, Nunclon™ Delta Surface) at 37°C in a 5% CO_2_ incubator. For immunocytochemistry, HEK293T cells were plated onto poly-l-lysine (Sigma^®^) coated coverslips.

### 2.9 Transfection

HEK293T cells were transiently transfected with plasmid DNA [for sequences see Murtha et al. (62)] encoding the following: mCherry (mCh), GFP, oncomodulin-mCh (OCM-mCh), or OCM-GFP. HEK293T cells were seeded to be 70% confluent at transfection. Transfection was performed using Lipofectamine™ 3,000 (Invitrogen™) in Opti-MEM™ (Gibco™, reduced serum medium) according to the manufacturer’s protocol.

### 2.10 MTS assay

To determine the LD_50_ of staurosporine (STS) (Tocris Bioscience™, 1 mM stock in Dimethyl Sulfoxide, DMSO), HEK293T cells were plated and transfected with the mCh plasmid (control) in 96-well plates (Thermo, Nunclon™ Delta Surface). 24-h post transfection, STS (5 × 10^−3^ μM – 10 μM in DMEM) was administered at various concentrations. 30% DMSO (MP Biomedicals) was used as a positive control for cell death. After 24 h, the DMEM media was exchanged for FluoroBrite™ DMEM. MTS reagent solution (Abcam, ab197010) was added according to the manufacturer’s protocol and incubated for 2 h at 37°C in a 5% CO_2_ incubator. 96-well plates were brought to room temperature, shaken briefly, and absorbance was measured at 490 nm using a xMark™ (Bio-Rad) microplate spectrophotometer. The optimal dose of thapsigargin (Tg) (Invitrogen™, 10 mM stock in DMSO), was determined through review of previous literature (69).

### 2.11 Confocal microscopy

All cytochemical stains were visualized using a Zeiss LSM800 upright confocal laser scanning microscope. Microdissected cochlear epithelia were imaged using a 10× air objective (N.A. 0.3) at 0.5× zoom to capture entire pieces. Then, higher magnification images were taken using a 40× oil immersion objective (N.A. 1.2). Z-stacks (2 μm apart) were taken to capture whole hair cells, using Hoechst, Phalloidin, and Myo7a to determine start and stop points. Mitochondria were visualized using Airyscan imaging under a 63x oil immersion objective (N.A. 1.4). Z-stacks (0.13 μm apart) were taken and subject to high Airyscan processing before analyzing. For confocal imaging of HEK293T mitochondria, single 63× Airyscan images were taken and processed. HEK293T TUNEL staining was imaged using a 20x air objective (N.A. 0.8).

### 2.12 Image and data analysis

ABR and DPOAE threshold data was transferred to Prism 10 (GraphPad) for statistical analysis and data visualization. A blinded researcher determined % OHC loss by counting the total number of OHCs present and missing in each 40× image. Missing OHCs were determined by loss of Myo7a/Phalloidin labeling and/or loss of nuclear staining. For TUNEL staining, the number of surfaces that were TUNEL positive was counted using the Surfaces feature in Imaris (Oxford Instruments). The number of TUNEL positive surfaces was normalized to a 1,000 μm^2^ OHC region, which corresponded to approximately 70–80 OHCs (in control animals). Confocal z-stacks from mid-modiolar cochlear sections were Airyscanned, processed and mitochondrial morphology of OHCs was analyzed using Imaris (Oxford Instruments) and FIJI. HEK293T cells were also analyzed for their mitochondrial morphology using FIJI plugins “Skeletonize” (70) and “Tubeness” (71). For a detailed description of mitochondrial morphology analysis (see Supplementary methods).

Four independent trials of noise exposure conducted on cohorts of 6–17 mice were performed. Each cohort was made up of both *Ocm* WT and KO mice which were either exposed to noise (106 dB SPL for 2 h) or used as controls/unexposed (kept in a sound-proof both for the duration of the exposure). Following noise-exposure, ears were harvested; for the majority of the experiments, left ears were used for microdissections (TUNEL and OHC counts) and right ears were sectioned and used for immunostaining (TOM20 and COXIV). To avoid inter-run variability in immunofluorescence (IF) staining, cochleae were frozen (in 100% glycerol) until use and IF staining was performed on the same day in 2 batches for each staining paradigm (i.e., TUNEL, TOM20, and COXIV). For any quantitative measures dependent on fluorescence intensity (TOM20 intensity, mitochondrial size, COXIV intensity), confocal imaging parameters and laser settings were kept consistent for both batches.

### 2.13 Statistics

All data was analyzed in Prism 10 (GraphPad). Unless otherwise stated, functional hearing assessments, including ABRs and DPOAEs, OHC loss, TUNEL staining, and mitochondrial morphology were analyzed for significance by Two-way ANOVA followed by a Tukey’s or Bonferroni’s multiple comparison’s test. The mean TOM20 and COXIV fluorescence intensity was compared using a Two-way ANOVA followed by a Sidak’s or Tukey’s multiple comparisons test. For *in vitro* mitochondrial morphology, a Kruskal–Wallis test, followed by a Dunn’s multiple comparisons test was used. The HEK293T TUNEL assay was analyzed using an unpaired *t*-test with Welch’s correction. All data is presented as mean values ± the standard error of the mean (S.E.M.) unless otherwise stated.

## 3 Results

### 3.1 *Ocm* KO mice are sensitive to noise-induced hearing loss

**Figure 1:**
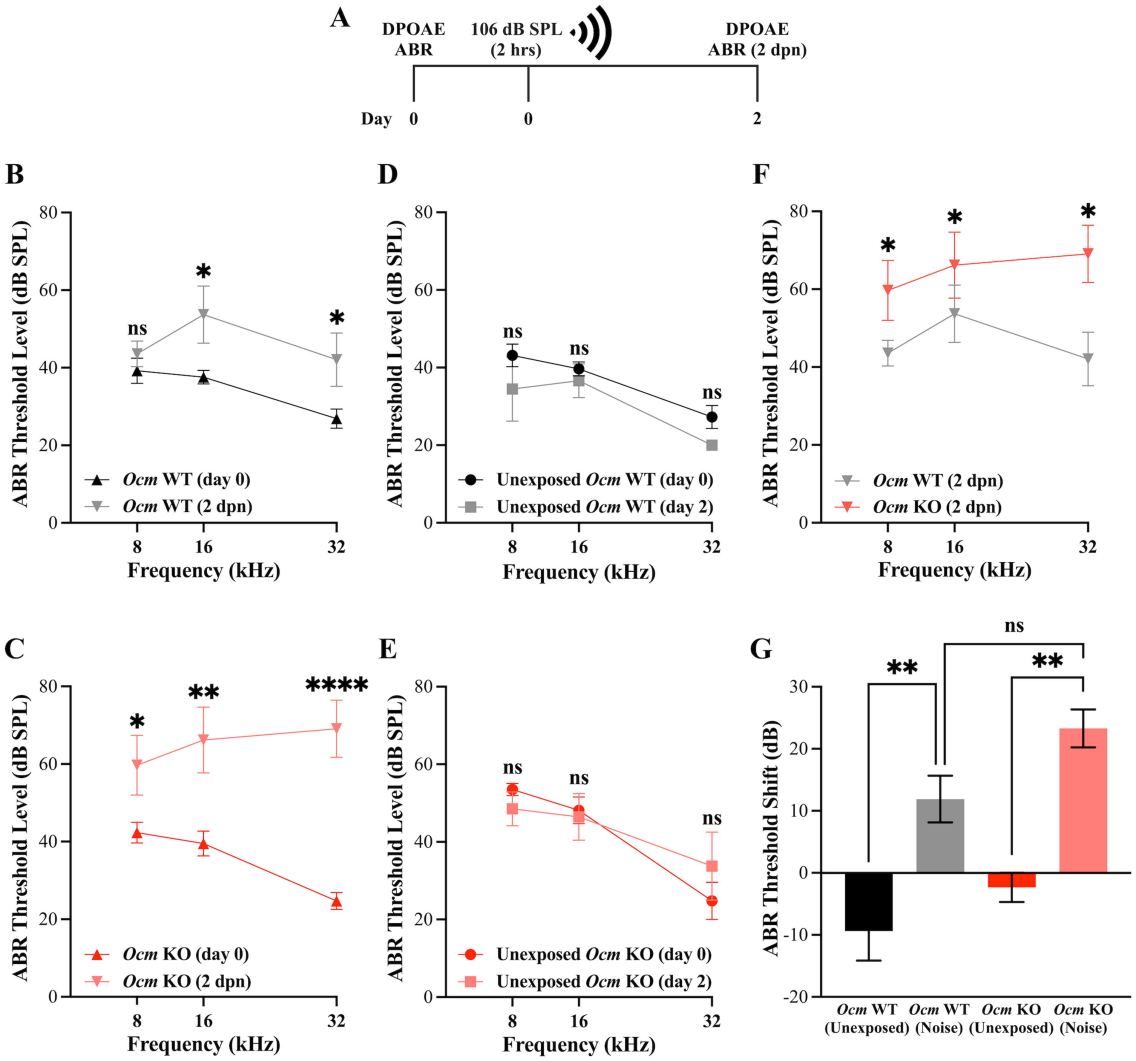
*Ocm* KO mice have higher ABR threshold levels compared to WT mice post-noise exposure. **(A)** Timeline of noise-exposure experiments. Three-week-old *Ocm* WT and *Ocm* KO mice were exposed to broadband noise at 106 dB SPL for 2 h. DPOAE and ABR measurements were taken prior to, and 2 days post-noise exposure (dpn). **(B,C)** ABR threshold levels at 8, 16 and 32 kHz pre-and 2 days post-noise exposure in *Ocm* WT **(B)** and *Ocm* KO **(C)** mice. **(D,E)** ABR threshold levels remain unchanged in control WT and KO mice. **(F)** Comparison of ABR threshold levels at 8, 16, and 32 kHz in *Ocm* WT and *Ocm* KO mice 2 days post-noise exposure (Two-way ANOVA with Bonferroni’s *post hoc*). **(G)** Average ABR threshold shift across all tested frequencies in control and noise exposed *Ocm* WT and *Ocm* KO mice. *n* ≥ 3 per group. All plotted values represent mean ± SEM. Asterisks represent statistical significance with: *p ≤ 0.05, **p ≤ 0.01, ***p ≤ 0.001, ****p < 0.0001, ns, not significant.

**Figure 2:**
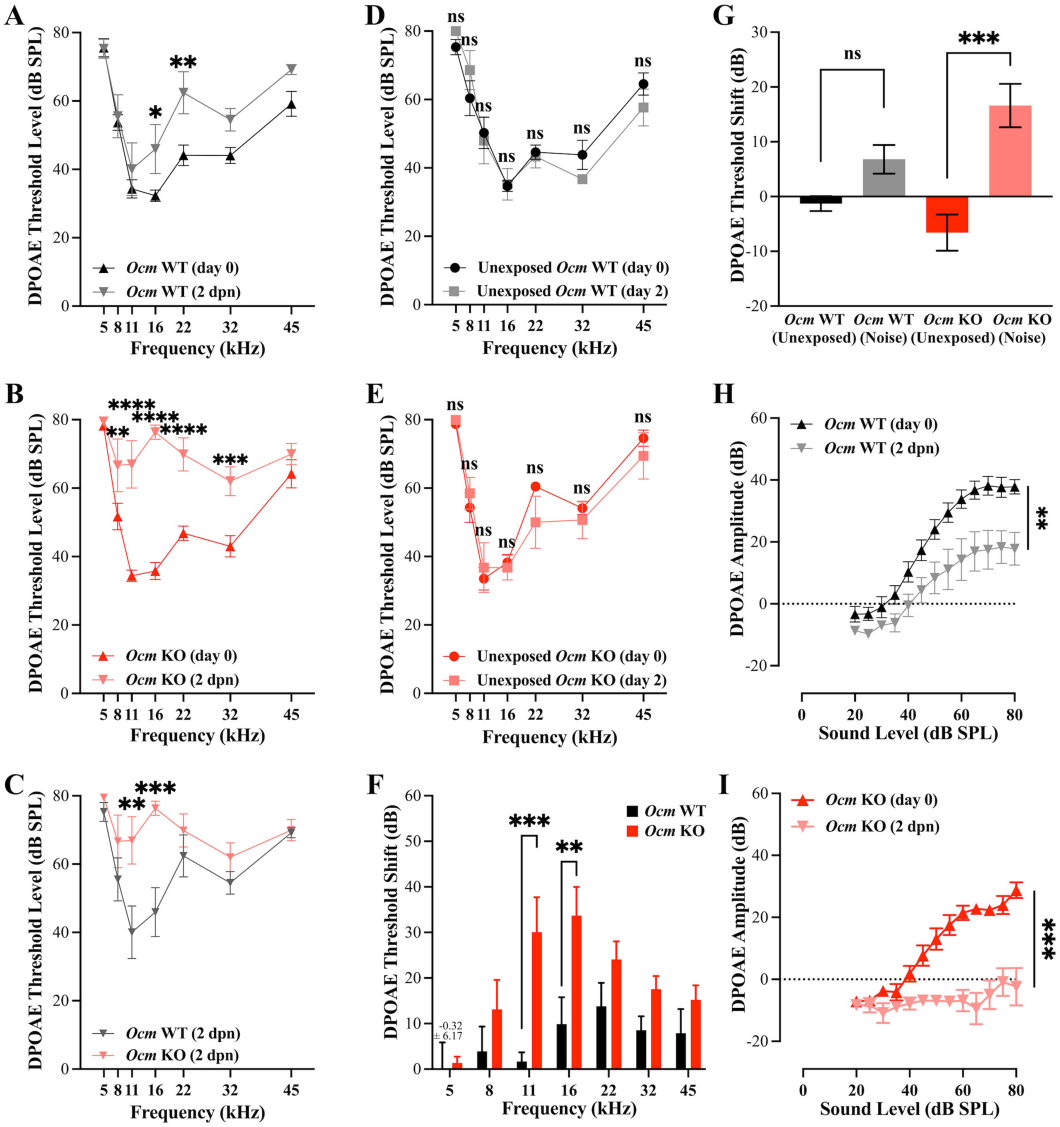
DPOAE threshold levels and I/O functions are higher in *Ocm* KO mice compared to WT mice post-noise exposure. **(A–E)** DPOAE threshold levels at 5, 8, 11, 16, 22, 32, and 45 kHz pre-and post-noise exposure in *Ocm* WT **(A)** and *Ocm* KO **(B)** mice **(C)** Comparison of DPOAE threshold levels in *Ocm* WT and *Ocm* KO 2 days post-noise exposure. **(D,E)** DPOAE threshold levels remain unchanged in control WT and KO mice. **(F)** Average DPOAE threshold shift at 5, 8, 11, 16, 22, 32, and 45 kHz 2 days post-noise exposure in *Ocm* WT and *Ocm* KO mice. **(G)** Average DPOAE threshold shift across all tested frequencies pre-and 2 days post-noise exposure in *Ocm* WT and *Ocm* KO mice. **(H,I)** DPOAE I/O at 16 kHz pre-and post-noise exposure in *Ocm* WT **(H)** and *Ocm* KO **(I)** mice *n* = ≥3 per group. All plotted values represent mean ± SEM. Asterisks represent statistical significance with: *p ≤ 0.05, **p ≤ 0.01, ***p ≤ 0.001, ****p < 0.0001, ns, not significant.

In previous studies, we reported that genetic deletion of *Ocm* reduces hearing over the mouse lifespan by about 50% compared to wildtype controls, irrespective of genetic background (25). Data from a companion study (64) suggests that moderate noise exposure (94.6 dB SPL) leads to a temporary elevation in hearing thresholds of *Ocm* WT mice with recovery after 2 weeks while age-matched *Ocm* KO mice showed no recovery. In the present study, we exposed 3–5-week-old *Ocm* WT and KO mice to broadband noise at 106 dB SPL for 2 h (Figure 1A). In order to investigate the dynamic period following noise, we collected DPOAEs and ABRs prior to and 2-days-post noise exposure (2 dpn). After noise exposure, ABR threshold levels were significantly (*p* = 0.0038) elevated at 16 kHz and 32 kHz in both *Ocm* WT and *Ocm* KO animals (Figures 1B,C) while control animals, not exposed to noise, remained unchanged (Figures 1D,E). Importantly, noise exposed *Ocm* KO animals had significantly (*p* = 0.0047) higher ABR threshold levels compared to noise exposed *Ocm* WT at all frequencies tested (Figure 1F). While the average total ABR threshold shift at all frequencies tested after noise exposure was seemingly higher in the *Ocm* KO animals compared to *Ocm* WT, this difference was not statistically significant (*p* = 0.0648) (Figure 1G). Following this, we measured DPOAEs, an indicator of OHC function, before and after noise exposure in *Ocm* WT and *Ocm* KO mice. DPOAE threshold levels were significantly (*p* < 0.001) elevated at 11 and 16 kHz in *Ocm* WT mice 2 days after noise exposure (Figure 2A). Comparatively, *Ocm* KO mice had significantly (*p* < 0.0001) elevated DPOAE threshold levels at 8, 11, 16, 22, and 32 kHz 2 days post-noise exposure (Figure 2B). DPOAE threshold levels in noise exposed *Ocm* WT and *Ocm* KO animals were statistically significant (*p* = 0.0002) at 11 and 16 kHz (Figure 2C). DPOAE threshold levels were unchanged in unexposed *Ocm* WT and KO animals over the course of 2 days (Figures 2D,E). Subsequent comparison of DPOAE threshold shifts at individual frequencies revealed a statistically significant (*p* < 0.0001) difference between *Ocm* WT and *Ocm* KO animals at 11 and 16 kHz after noise exposure (Figure 2F). While average total DPOAE threshold shifts across all tested frequencies were significantly higher in *Ocm* KO mice after noise exposure (*p* = 0.0004, Kruskal–Wallis test, uncorrected Dunn’s test), average total threshold shifts were not significantly different before and after noise exposure in *Ocm* WT mice (*p* = 0.0512) (Figure 2G). Furthermore, DPOAE input/output functions were assessed at 16 kHz in *Ocm* WT and *Ocm* KO mice pre-noise and 2 days post-noise exposure. In *Ocm* WT mice, input/output functions shifted to the right 2 days post-noise exposure (Figure 2H). However, *Ocm* KO mice after noise exposure lacked suprathreshold responses, indicative of an absence of DPOAEs (Figure 2I). These data suggest that *Ocm* KO mice are more sensitive to noise damage from the parameters used in this study (106 dB SPL for 2 h). Additionally, these findings indicate an essential function of the Ca^2+^ buffer, OCM, in protecting against NIHL, similar to its role in delaying early progressive hearing loss (25, 31).

### 3.2 *Ocm* KO mice exhibit increased outer hair cell loss compared to WT mice

**Figure 3:**
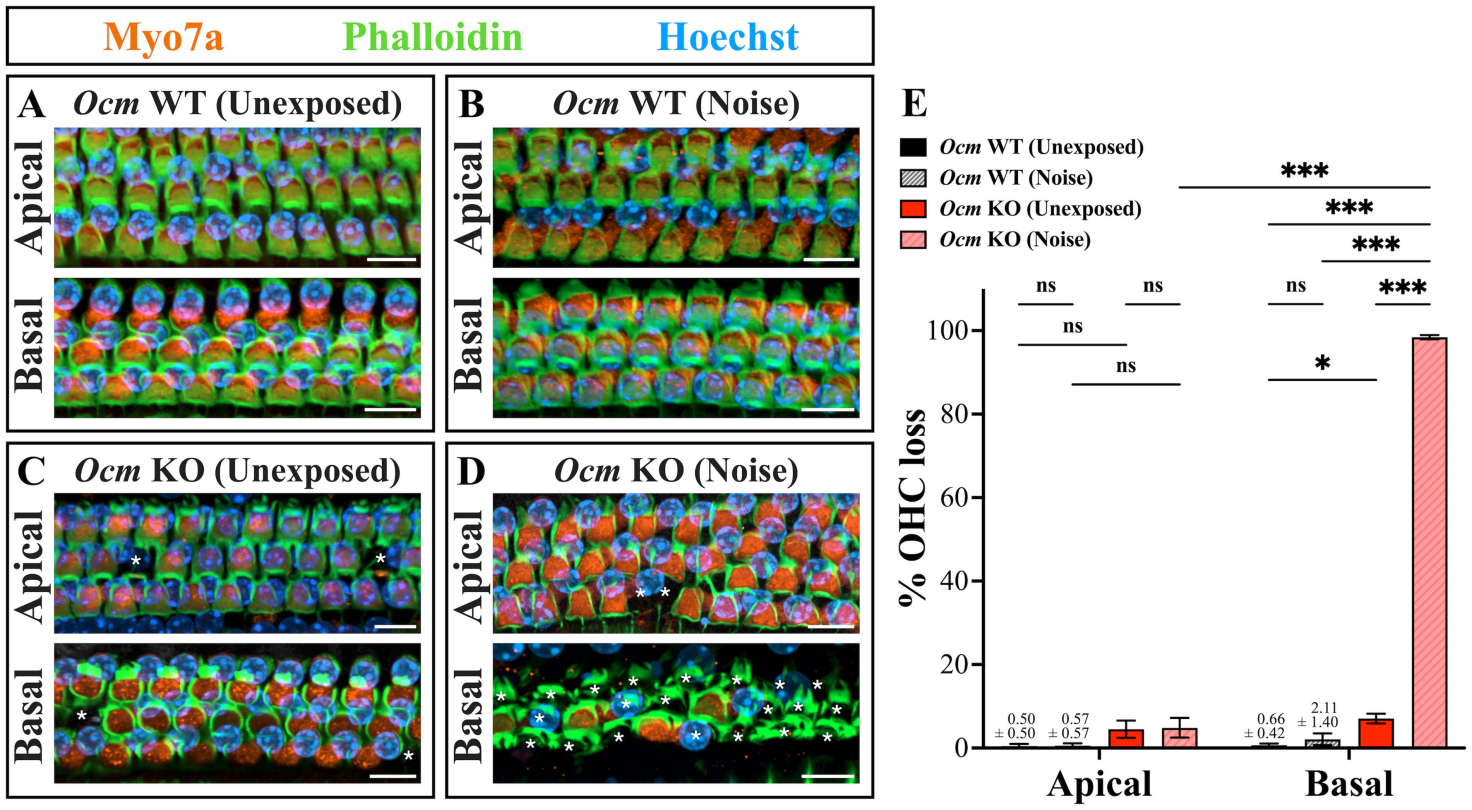
*Ocm* KO mice are more prone to outer hair cell loss compared to *Ocm* WT mice. **(A–D)** Representative images of cochlear whole mounts in the apical (~8–12 kHz) and basal (~28–48 kHz) frequency regions. Orange = Myo7a, green = Phalloidin, blue = Hoechst. 40× maximum intensity projections (MIPs). White asterisks denote missing OHCs. Scale bar = 10 μm. Shown are images from *Ocm* WT control **(A)**, *Ocm* WT noise **(B)**, *Ocm* KO control **(C)**, and *Ocm* KO noise exposed mice **(D)**. **(E)** Quantification of OHC loss in *Ocm* WT and KO control and noise exposed mice. *n* ≥ 5 per group. All plotted values represent mean ± SEM. Asterisks represent statistical significance with: *p ≤ 0.05, ***p ≤ 0.001; ns, not significant.

Noise exposure rapidly induces elevated cytoplasmic Ca^2+^ levels in OHCs (49), which has been linked to apoptotic hair cell death (48, 72, 73). We hypothesized that loss of OCM would leave OHCs particularly susceptible to noise induced-death due to elevated levels of Ca^2+^ within 1–2 days following noise exposures. To investigate this, cochlear whole mounts were prepared from unexposed and noise-exposed *Ocm* WT and KO mice and OHC loss quantified in the apical (~8–12 kHz) and basal (~28–48 kHz) regions. Cells were considered present if they had positive Myo7a, Phalloidin and Hoechst labeling (Figures 3A–D). In both *Ocm* WT and KO cochlea without noise exposure, there was little or no OHC loss in the apex and base (Figures 3A–E). After noise exposure, *Ocm* WT OHCs in the apical regions exhibited no OHC loss while there was some degree of OHC loss in the base which was not significantly different from control *Ocm* WT cochlea (Figures 3B,E). After noise exposure, *Ocm* KO OHCs had a far greater degree of OHC loss in the basal region compared to unexposed KO OHCs. In some cases, basal regions in the *Ocm* KO cochlea had nearly 100% OHC loss. However, similar to the WTs, there was no statistically significant difference in the apical regions of noise-exposed *Ocm* KO OHCs compared to unexposed KO OHCs (Figures 3D,E). Overall, the noise exposure parameters used in this study produced little or no OHC loss in WT mice, but exacerbated OHC loss in *Ocm* KO mice especially in higher frequency regions.

### 3.3 Increased TUNEL labeling was detected in noise exposed *Ocm* KO OHCs

**Figure 4:**
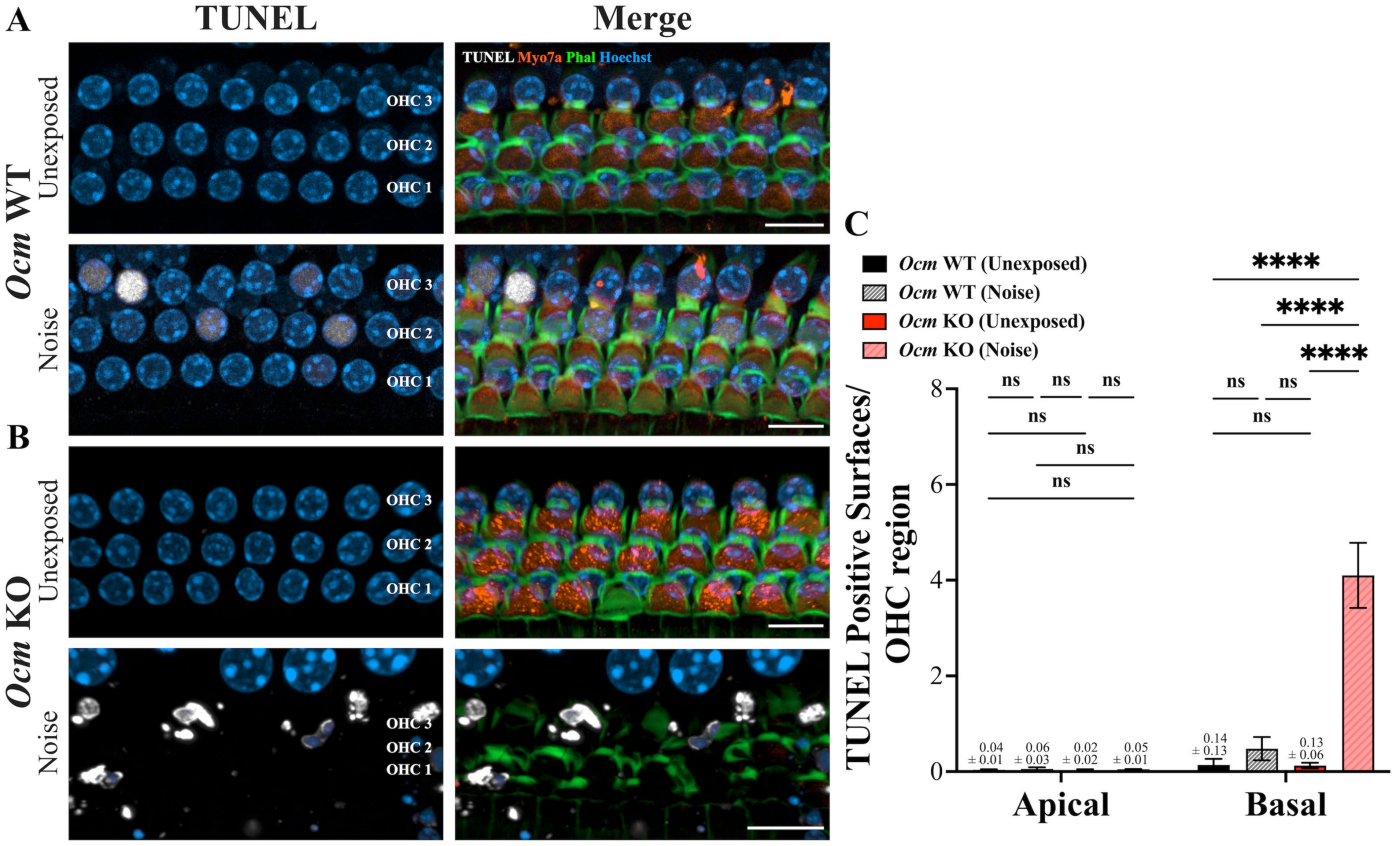
TUNEL staining is increased in noise-exposed *Ocm* KO mice compared to WT mice. **(A,B)** Representative MIPs of confocal z-stacks from control and noise exposed *Ocm* WT and KO basal OHCs. Cochlea were harvested 1 day post noise exposure. Three rows of OHCs are shown. White = TUNEL, blue = Hoechst, orange = Myo7a, green = Phalloidin. Scale bar = 10 μm. **(C)** Bar graphs represent the number of TUNEL positive surfaces per OHC region. *n* ≥ 5 per group. All plotted values represent mean ± SEM. Asterisks represent statistical significance with: ****p < 0.0001; ns, not significant.

In noise-exposed GCaMP6s mice, *Ocm* KO mice had substantially more OHC loss than WT mice, suggesting that the absence of OCM may make mutant OHCs more vulnerable to cell death. To quantify the degree of DNA fragmentation, a hallmark of apoptosis, prior to or following noise exposure, we used a TUNEL assay (74). We harvested tissue from unexposed and noise-exposed *Ocm* WT and KO mice 24 h post noise exposure and performed TUNEL staining (Figures 4A,B). We quantified the amount of DNA fragmentation in the OHC region of unexposed and noise exposed *Ocm* WT and KO mice (Figure 4C). There was little evidence of TUNEL staining in apical or basal OHC regions of unexposed *Ocm* WT and KO cochleae. However, there was increased TUNEL staining in the basal region of both *Ocm* WT and KO OHCs following noise exposure (Figure 4C). In the WT cochleae, this increase after noise exposure was not significantly different from unexposed *Ocm* WT cochleae (Figure 4C). The greatest amount of TUNEL staining was observed in the basal region of *Ocm* KO OHCs after noise exposure and was significantly greater than unexposed *Ocm* KO OHCs (Figure 4C). These results further suggest that OHCs of *Ocm* KO mice are especially vulnerable to cell death after noise damage.

### 3.4 Loss of OCM alters mitochondrial morphology in OHCs

**Figure 5:**
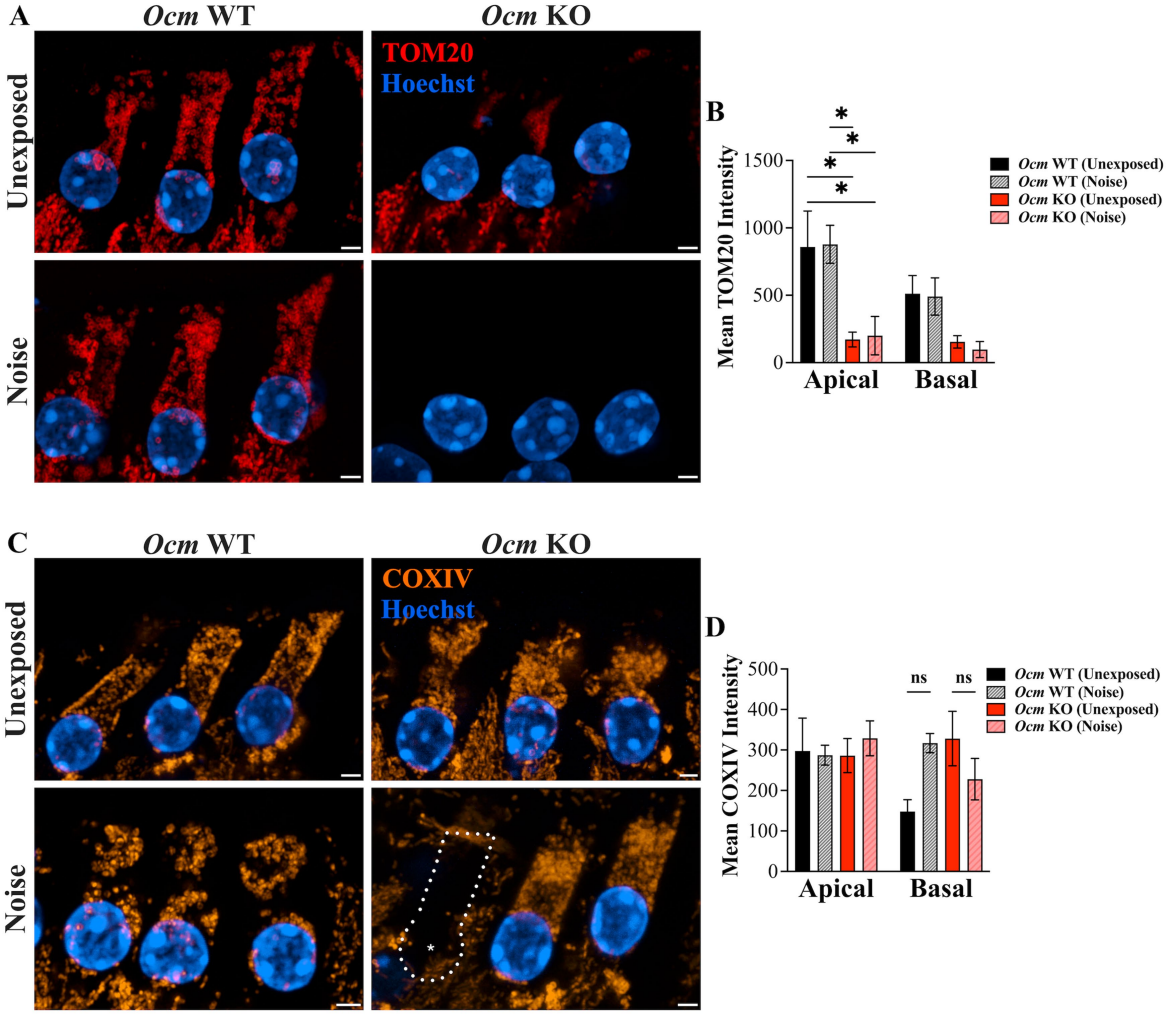
TOM20 and COXIV show different expression patterns in apical and basal OHCs of unexposed and noise exposed *Ocm* WT and KO mice. **(A–C)** state Representative MIPs of basal OHCs are shown. **(A)** TOM20 immunostaining in OHCs. red = TOM20, blue = Hoechst. Scale bar = 2 μm. **(B)** Quantification of TOM20 fluorescence intensity. *n* ≥ 3 per group. **(C)** COXIV immunostaining in OHCs. orange = COXIV, blue = Hoechst. Scale bar = 2 μm. **(D)** Quantification of COXIV fluorescence intensity. *n* = 2–4 per group. All plotted values represent mean ± SEM. Asterisks represent statistical significance with: *p ≤ 0.05, ****p < 0.0001; ns, not significant.

Mitochondrial-mediated apoptosis has been implicated in OHC death (48, 72, 75–81). This prompted us to examine the mitochondria of unexposed and noise exposed *Ocm* WT and KO OHCs. TOM20 and COXIV were used as mitochondrial markers (Figures 5A,C). TOM20 labeling was observed across all three rows of OHCs of control and noise exposed *Ocm* WT cochlea (Figure 5A). In contrast, both control and noise exposed *Ocm* KO OHCs had sparse TOM20 labeling (Figure 5A). To quantify this, we measured mean intensity of TOM20 in apical and basal OHCs (Figure 5B). TOM20 fluorescence intensity was significantly lower (*p* = 0.0015, Two-way ANOVA) in apical noise exposed *Ocm* KO OHCs (200.36 ± 142.18) compared to both *Ocm* WT control (unexposed: 858.28 ± 267.03) and noise exposed (511.92 ± 134.88) OHCs. Surprisingly, unexposed *Ocm* KO OHCs in apical regions also had significantly (*p* = 0.406) lower TOM20 fluorescence intensity values compared to apical control OHCs (unexposed *Ocm* WT). Next, we measured COXIV fluorescence intensity (Figure 5D). COXIV fluorescence intensity exhibited a slightly different pattern. Although there was no statistical significance (Two-way ANOVA), in noise-exposed basal *Ocm* WT OHCs, there was a trend toward increased COXIV fluorescence intensity compared to unexposed animals. In basal *Ocm* KO OHCs, the trend was the opposite; where unexposed OHCs had increased COXIV intensity compared to noise exposed OHCs, although this did not reach significance either. The differences in immunostaining could be attributed to differences in ages between the TOM20 and COXIV immunostaining, where the *Ocm* KO animals used for TOM20 expression were 3–5-weeks-of-age and the *Ocm* KO animals used for COXIV expression were only 3–4 weeks-of-age.

**Figure 6:**
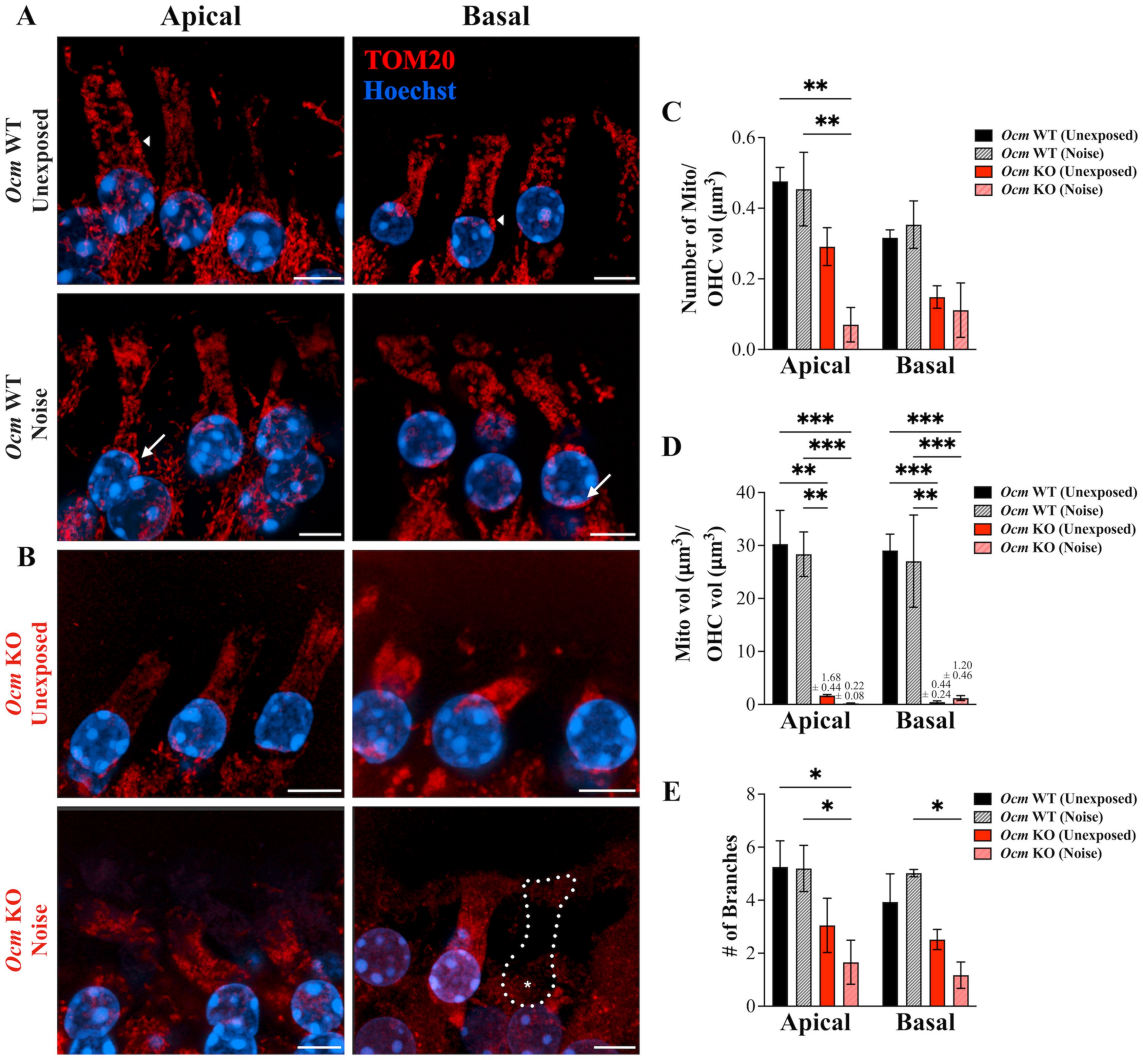
Mitochondrial morphology is altered in control and noise-exposed *Ocm* KO OHCs. **(A,B)** Apical and basal OHCs from mid-modiolar sections of unexposed and noise exposed *Ocm* WT and KO cochleae harvested 2 dpn. Representative MIPs are shown. Red = TOM20, blue = Hoechst. Scale bar = 5 μm. All images were taken using the same imaging parameters. However, brightness/contrast display settings were optimized for each representative image shown here for visualization purposes. Dotted outlines with asterisks denote missing OHCs. Arrows highlight areas of mitochondrial aggregation along the lateral membrane (short arrows) and in the perinuclear region (long arrows) in *Ocm* WT unexposed and noise exposed, respectively. **(C)** Bar graph showing the number of mitochondria/OHC volume (μm^3^), **(D)** mitochondrial volume (μm^3^)/OHC volume (μm^3^), and **(E)** the mean number of branches per mitochondria in apical and basal OHCs of control and noise exposed *Ocm* WT and KO OHCs. *n* ≥ 3 per group. All plotted values represent mean ± SEM. Asterisks represent statistical significance with: *p ≤ 0.05, **p ≤ 0.01, ***p ≤ 0.001, ****p < 0.0001; ns, not significant.

Studies suggest mitochondria change size and localization in OHCs in response to ototoxic drugs and noise exposure (82, 83). Throughout aging, OHC mitochondria reduce in number and exhibit structural pathology (47). Thus, we hypothesized that deletion of the primary Ca^2+^ buffer OCM would alter mitochondrial morphology. We used TOM20, which is considered a reliable marker to visualize the mitochondrial network (84, 85), to quantify abundance, volume, and branching of mitochondria in OHCs from GCaMP6s *Ocm* WT and KO mice both unexposed and exposed to noise (Figures 6A,B). In order to display mitochondrial morphology, the brightness and contrast settings were altered between groups for this figure only. The mean number of mitochondria per OHC volume (μm^3^) was significantly reduced (*p* = 0.0002) in apical noise exposed *Ocm* KO OHCs (0.070 ± 0.049) compared to both unexposed (0.476 ± 0.039) and noise exposed (0.454 ± 0.104) *Ocm* WT OHCs (Figure 6C). A similar decrease in mitochondrial abundance was observed in the basal region, however, pairwise comparisons did not significantly differ (*p* = 0.0525, Two-way ANOVA, Tukey’s multiple comparisons). Next, mitochondrial size, as measured by mitochondrial volume (μm^3^) over OHC volume (μm^3^) was calculated (Figure 6D). In both apical and basal regions, noise exposed *Ocm* KO OHCs had a lower mitochondrial volume per OHC volume (apical: 0.213 ± 0.077; basal: 1.198 ± 0.458) compared to control (*Ocm* WT unexposed; apical: 30.27 ± 6.35; basal: 29.07 ± 3.06). Surprisingly, *Ocm* KO OHCs not exposed to noise also displayed smaller mitochondrial volumes (*Ocm* KO unexposed; apical: 1.68 ± 0.187; basal: 0.441 ± 0.236) compared to controls (*Ocm* WT unexposed OHCs). Next, the mean number of mitochondrial branches was measured (Figure 6E). In apical regions, noise exposed *Ocm* KO OHCs had fewer number of mitochondrial branches (1.66 ± 0.831) compared to both unexposed (5.26 ± 0.984) and noise exposed (5.20 ± 0.871) *Ocm* WT OHCs. In basal regions, the number of mitochondrial branches only significantly differed (*p* = 0.0007, Two-way ANOVA) between noise exposed *Ocm* WT (5.02 ± 0.138) and KO (1.17 ± 0.500) OHCs (Tukey’s multiple comparisons). In general, mitochondria from *Ocm* KO OHCs appeared more punctate and lacked organization compared to *Ocm* WT OHCs. Additional measures of mitochondrial morphology including sphericity, distance to nearest neighbor, ellipticity (oblate and prolate) were quantified for each group, but no significant differences were found (Two-way ANOVA) (Supplementary Figure S1). Overall, no significant differences in values of mitochondrial morphology measured were found between control (unexposed) and noise exposed *Ocm* WT OHCs (Tukey’s multiple comparisons). However, we did observe aggregation of mitochondria around the perinuclear region of noise exposed *Ocm* WT OHCs (Figure 6A, white arrows). In summary, *Ocm* WT OHCs maintain normal mitochondrial morphology 2 days post noise exposure but may exhibit patterns of redistribution, while *Ocm* KO OHCs have smaller, fragmented mitochondria.

### 3.5 OCM expression protects against Ca^2+^-induced stress in an HEK293T cell culture model

**Figure 7:**
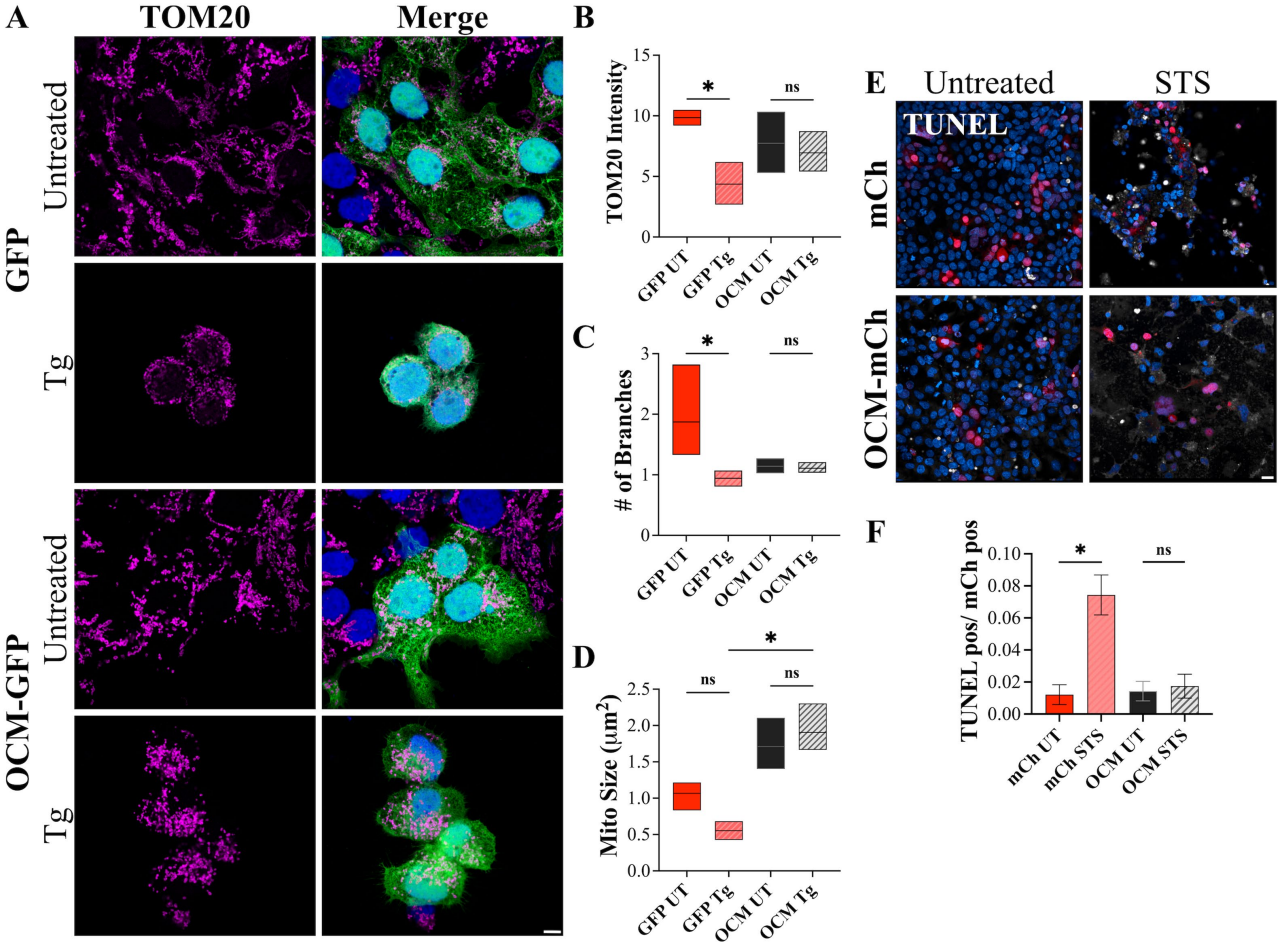
HEK293T cells expressing OCM are less vulnerable to Ca^2+^-related apoptotic inducers. HEK293T cells were transiently transfected with GFP (control) or OCM-GFP and incubated with thapsigargin (Tg, 1 μM) for 48 h. **(A)** Representative Airyscan images of control (untreated) and Tg treated GFP and OCM-GFP expressing cells. Magenta = TOM20, blue = Hoechst. Scale bar = 5 μm. Mitochondrial morphology was measured by calculating **(B)** the mean (integrated density) TOM20 intensity, **(C)** number of mitochondrial branches, and **(D)** the average mitochondrial size (μm^2^). Data is shown as a floating bar graph (min to max) with a line at the mean. *n* = 3 technical replicates. A TUNEL assay was performed using staurosporine treated (STS, 24 h) or untreated HEK293T cells transiently expressing mCh (control) or OCM-mCh. **(E)** Shown are representative confocal images from these experiments. TUNEL = white, blue = Hoechst. Scale bar = 20 μm. **(F)** The bar graph shows mean number of TUNEL positive cells over total mCh positive cells. *n* = 3 experimental replicates. All plotted values represent mean ± SEM. Asterisks represent statistical significance with: *p ≤ 0.05; ns, not significant.

The OCM appears to have a protective effect on OHCs against cellular stress, particularly with Ca^2+^ related stress. To see if OCM protects against Ca^2+^-induced cell death in a more general context, we utilized a mammalian cell culture model. Thapsigargin (Tg) is an inhibitor of the SR/ER Ca^2+^ ATPase (SERCA) protein, which transports Ca^2+^ from the cytoplasm into the ER/subsurface cisternae (SSC). Previous studies show that Tg induces mitochondrial fragmentation and apoptosis through prolonged exposure to high levels of intracellular Ca^2+^ (Horn et al., 2007). Therefore, we exposed HEK293T cells expressing GFP-tagged OCM (OCM-GFP) or GFP (control) to 48 h of Tg treatment. Then, we used TOM20 to visualize mitochondrial morphology (Figure 7A). With Tg administration, GFP+ cells had reduced TOM20 staining intensity (4.369 ± 1.016) compared to untreated cells (9.85 ± 0.648) (Figure 7B). However, HEK293T cells transfected with OCM-GFP showed no reduction in TOM20 intensity when administered Tg. Similarly, the number of mitochondrial branches for Tg-treated GFP cells was lower (0.943 ± 0.0767) compared to untreated GFP+ cells (1.87 ± 0.476) (Figure 7C), while the number of branches for OCM-GFP HEK293T cells was unchanged with Tg administration. Finally, the average size of mitochondria (μm^2^) was significantly lower (*p* = 0.0006, Kruskal–Wallis test) for control Tg-treated HEK293T cells expressing GFP only (0.554 ± 0.0742) compared to cells expressing OCM-GFP (1.91 ± 0.201) (Figure 7D). We also tested to see if the effects of another Ca^2+^ related inducer of apoptosis, staurosporine (STS), would be ameliorated by the expression of OCM. The LD_50_ of STS for 24 h of incubation was determined to be 0.0829 μM by an MTS assay (Supplementary Figure S2). STS was administered to HEK293T cells expressing mCh (control) or mCh-tagged OCM for 24 h (Figure 7E). The number of TUNEL positive cells (over mCh positive cells) in STS-treated control HEK293T cells (0.0744 ± 0.0125) was significantly higher (*p* = 0.0219, Welch’s *t-*test) than mCh control (mCh only: 0.0122 ± 0.00619) (Figure 7F). On the contrary, STS administration to OCM-expressing HEK293T cells did not alter the number of TUNEL positive cells. These experiments indicate that transfection of OCM rescues mitochondrial morphology altered by prolonged Tg administration and mitigates STS-induced apoptosis, implying that OCM may have protective effects against Ca^2+^-induced cell death.

## 4 Discussion

Calcium buffering plays a major role in the sensitivity of OHCs to injury and loss following noise exposure. The ability of OHCs to regulate cytosolic Ca^2+^ levels depend not only on mobile protein Ca^2+^ buffers such as OCM, but also on organelles such as mitochondria. Previous studies show that targeted deletion of *Ocm* leads to an early progressive hearing loss phenotype and prehearing alterations in Ca^2+^ signaling (23, 31, 62). The results from the present study demonstrate, that in the absence of OCM, OHCs become more vulnerable to noise-induced dysfunction, damage and loss. We compared noise-induced alterations in *Ocm* WT and KO mice 48 h after noise exposure (106 dB SPL, 2 h). After noise exposure, *Ocm* KO mice had significantly higher ABR and DPOAE threshold shifts. In addition, *Ocm* KO mice had more OHC loss, especially in basal regions of the cochlea compared to *Ocm* WT mice, both with, and without noise exposure. The higher level of OHC loss was complemented by alterations in OHC mitochondrial morphology. In GCaMP6s mice, we found that *Ocm* KO OHCs had decreased mitochondrial abundance, lower mitochondrial volume, decreased branching, and lower TOM20 fluorescence intensity. In our mammalian cell culture model, we show that OCM has protective effects against compounds that induce mitochondrial damage and apoptosis. We conclude that alterations in Ca^2+^ buffering, via genetic deletion of *Ocm*, leads to deleterious morphological and functional changes to OHCs.

### 4.1 Calcium homeostasis in OHCs

Ca^2+^ homeostasis in OHCs is coordinated through numerous Ca^2+^-related proteins forming a regulatory network, balancing Ca^2+^ entry, extrusion and free cytoplasmic levels. The main sources of Ca^2+^ entry into OHCs are MET channels at the tips of the stereocilia and voltage-gated Ca^2+^ channels (Ca_v_1.3) along the basolateral membrane (86–88). Deletion of Ca_v_1.3 (*Ca_v_1.3^−/−^*) leads to early loss of OHCs beginning in apical regions (89–92). Extrusion of Ca^2+^ from OHCs is performed primarily by the plasmalemmal Ca^2+^ pump isoform 2 (PMCA2), which is localized to OHC stereocilia (93, 94). Mutations in *PMCA2* lead to OHC loss and progressive hearing loss (29, 95). Disruption of any of the key proteins involved in OHC Ca^2+^ homeostasis leads to OHC dysfunction, loss, and subsequent hearing loss. Here, we focus on the predominant CaBP of OHCs – OCM (37, 39). In the present study, using GCaMP6s mice, we found that *Ocm* KO mice had significantly elevated hearing thresholds after noise exposure and lacked suprathreshold DPOAE responses (at 16 kHz) compared to WT controls. Our previous reports of genetic deletion of *Ocm* show that these KO mice have an early, progressive hearing loss phenotype in multiple strains of mice (25, 31). Murtha et al. (62) reports that OCM significantly alters Ca^2+^ signaling early in development and decreases intracellular Ca^2+^ levels. Early postnatal disruptions in Ca^2+^ signaling by deletion of *Ocm*, which influences synaptic maturation and the expression of purinergic receptors (23), may also lead to alterations in the network of proteins and organelles that orchestrate Ca^2+^ signaling later on in life. Consistent with this, a companion study (64) shows that *Ocm* KO OHCs have larger ATP-induced Ca^2+^ transients compared to *Ocm* WT OHCs.

While OHCs from *Ocm* KO mice display membrane potentials and basolateral membrane currents that are similar to *Ocm* WT, loss of OCM disrupts normal Ca^2+^ buffering dynamics that play important roles in sculpting Ca^2+^ signaling and the development of spontaneous activity in OHCs (23, 62). Based on our previous findings, *Ocm* KO OHCs have higher levels of free cytosolic Ca^2+^ and higher maximum induced Ca^2+^ transients. Additionally, *Ocm* KO OHCs have an increased level of coordinated spontaneous Ca^2+^ activity compared to WT OHCs. In a companion study (64), we investigated whether the susceptibility of *Ocm* KO mice to prolonged (9 h) noise exposure was a result of increased Ca^2+^ signaling in OHCs. This study also showed that *Ocm* KO mice are not only more sensitive to NIHL, but unlike their WT counterparts, do not recover from moderate noise exposures (95 dB SPL). We found that prolonged noise stimulation caused an increase in maximum Ca^2+^ transients in OHCs from both *Ocm* WT and *Ocm* KO mice. However, OHCs from *Ocm* KO mice exhibited higher maximum Ca^2+^ transient signals after noise compared to *Ocm* WT mice. Comparisons of the maximum Ca^2+^ signals between pre-noise OHCs in *Ocm* KO mice and post-noise OHCs in *Ocm* WT mice were similar, suggesting that the absence of OCM mimics or creates a noise-exposed condition. Since Ca^2+^ overload in sensory hair cells contributes significantly to hair cell apoptotic and necrotic pathways, higher Ca^2+^ levels in *Ocm* KO OHCs might exacerbate these cell death pathways (49, 55, 96). Thus, tight regulation of intracellular Ca^2+^ signaling through specialized mobile CaBPs, such as OCM (66), may be essential for OHC survival, especially after noise exposure. Similar to studies of *Ocm* KO mice (23, 31, 62), genetic deletion of the *Mcu*, a mitochondrial Ca^2+^ influx channel, does not affect cochlear development or morphology of hair cells in the first two postnatal weeks (46). However, *Mcu* KO mice display high-frequency hearing loss by 3 weeks-of-age. By 3 months-of-age, *Mcu* KO mice have elevated ABR thresholds at all frequencies and loss of hair cell stereocilia. In line with previous studies of our *Ocm* KO mouse model, dysregulated Ca^2+^ signaling may not impair development, but eventually decreases OHC survival and leads to hearing loss.

### 4.2 Mitochondrial response to calcium imbalance

Since OCM plays a critical role in shaping Ca^2+^ responses in OHCs, the lack of a phenotype until young adult ages suggests that there are transient compensatory buffering mechanisms. In the present study, we investigated whether deletion of *Ocm* leads to alterations in mitochondria. Similar to mobile protein Ca^2+^ buffers, mitochondria act as specialized Ca^2+^ reservoirs, which spatiotemporally shape Ca^2+^ signaling (97, 98). Hair cells have a highly organized arrangement of mitochondria compared to neighboring supporting cells and their mitochondrial architecture develops during maturation (99). Transmission electron microscopy studies show that mitochondria localize to three distinct subcellular regions within OHCs: (1) along the lateral plasma membrane, (2) under the cuticular plate, and (3) surrounding the nucleus (41, 47). Our immunocytochemical analysis of WT OHCs falls in line with these observations. Previous studies have proposed that hair cells contain different subtypes of mitochondria, where mitochondria at the subcuticular plate may be more sensitive to damage and are unable to recover in the same capacity that perinuclear mitochondria can (47, 100). Our observations of increased TOM20 staining around the perinuclear region of noise-exposed WT OHCs could be a result of differences in resilience of mitochondrial subtypes. It is also possible that perinuclear mitochondria exhibit increased turnover rates, increased fission, or that mitochondria re-localize to the perinuclear region of the OHCs following noise-exposure. Mitochondrial motility has been heavily implicated in the maintenance of energy homeostasis and Ca^2+^ signaling in neurons (101, 102). Some of the proteins that make up the mitochondrial motility machinery (MIRO, SNPH) are capable of sensing local Ca^2+^ concentrations and regulating mitochondrial motility accordingly (103, 104). Mitochondria have been shown to delocalize from the lateral plasma membrane of OHCs during early apoptosis in response to ototoxic injury and exhibit degradation during late apoptosis (82). Additional work needs to be done to investigate mitochondrial dynamics following noise exposure in order to elucidate the process(es) that drive the change in mitochondrial subcellular organization observed here.

In this study, we observed decreased expression of TOM20 in *Ocm* KO OHCs compared to *Ocm* WT OHCs, particularly in basal regions of the cochlea. The decreased TOM20 labeling and smaller mitochondrial volume indicate mitochondrial degradation (105–108). Exposure of the inner mitochondrial membrane from the degradation of outer membrane proteins triggers mitophagy (109), which, in some cases, leads to apoptosis (110). Decreased mitochondrial abundance is thought to contribute to the vulnerability of hair cells to damage (111–113). Perkins et al. (47) showed that the number and size of mitochondria in OHCs decrease with age and mitochondria become depolarized in OHCs of 24-month-old mice (47). This study also showed that mitochondrial tethering to the SSC was decreased in aged OHCs. In another study, performed in postmortem human cochlea, it was found that aged patients with hearing loss had decreased expression of TOM20 in the organ of Corti (114). As age-related hearing loss in humans can be quantitatively predicted by hair cell loss, it is critical to better understand what mechanisms underlie OHC vulnerability.

**Figure 8:**
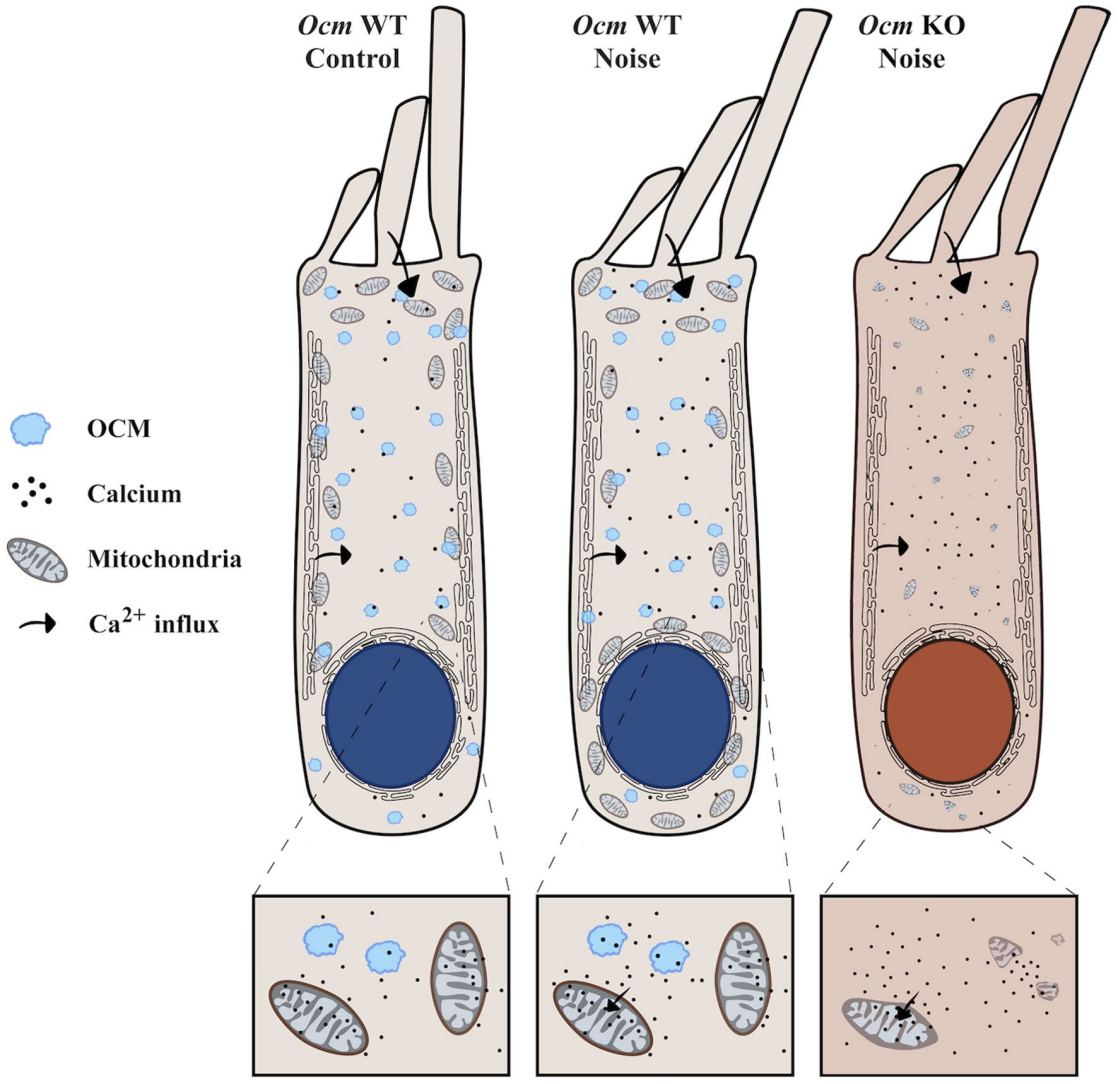
*Ocm* KO OHCs are predisposed to decreased mitochondrial abundance. The illustration represents our proposed model of altered mitochondrial dynamics in *Ocm* WT and KO OHCs exposed to noise. OCM is shown in light blue. Arrows indicate the movement of Ca^2+^ inside the OHCs. **(Left)** In unexposed *Ocm* WT OHCs, mitochondria display a characteristic organization pattern, clustering around the lateral plasma membrane and under the subsurface cisternae. **(Middle)** Following noise exposure in *Ocm* WT OHCs, we found that mitochondria aggregate along the perinuclear region. **(Right)** *Ocm* KO OHC mitochondria are predisposed to decreased mitochondrial volume, abundance and lack organization. Following noise exposure, mitochondria of *Ocm* KO OHCs become sparse and fragmented, leaving the cell vulnerable to OHC death.

We hypothesize a mechanism whereby loss of *Ocm* leads to prolonged Ca^2+^ overload and subsequent mitochondrial degradation, which leaves OHCs vulnerable to noise-induced OHC death (Figure 8). In other words, the presence of OCM is protective. However, while we observed decreased TOM20 staining in *Ocm* KO OHCs, which was associated with increased TUNEL staining, we do not present causal evidence linking decreased mitochondrial abundance to induction of cell death in *Ocm* KO OHCs. One limitation of this study is that we do not functionally evaluate mitochondria. Future work will include experiments to determine mitochondrial Ca^2+^ flux (e.g., Rhod-2 AM) and mitochondrial membrane potential (e.g., TMRM) (115). An alternative to our hypothesis is that a reduction in TOM20 protein expression could reflect a lower density of TOM20 in the outer mitochondrial membrane. Studies using stimulated emission depletion microscopy have shown that the density of TOM20 clusters is higher in more energetically active mitochondria (116). It is possible that the reduction in TOM20 protein expression in *Ocm* KO OHCs is due to decreased metabolic demand. This decrease may explain differences in patterns of fluorescence intensity between TOM20 and COXIV, which was unexpected, since TOM20 and COXIV expression levels typically correlate with one another (117). Another possible explanation for these differences could be the age ranges used for TOM20 and COXIV immunocytochemistry experiments, as age-related changes in both TOM20 and COXIV expression have been reported (47, 114, 118, 119). It would be interesting to compare TOM20 and COXIV expression patterns in *Ocm* KO OHCs at different ages.

Although reductions in the number and size of mitochondria and intensity of TOM20 staining were seen for *Ocm* KO OHCs in the basal regions, the values used to quantify mitochondrial morphology did not reach significance as they did in apical regions. This lack of significance was surprising and could reflect the methods used to quantify mitochondrial morphology in basal *Ocm* KO OHCs. Basal OHCs are more vulnerable to a wide variety of cellular insults including noise, ototoxic compounds, and aging. In this study, basal OHCs from *Ocm* KO mice exhibited the greatest loss following noise exposure, thus hindering accurate quantification of mitochondrial morphology. Future studies may include shorter durations or less intense noise exposure (<95 dB SPL) to observe more nuanced changes in *Ocm* KO OHCs. Alternatively, higher resolution techniques, like electron microscopy, which do not rely on mitochondrial markers, could be utilized to visualize damaged or fragmented mitochondria.

Our *in vitro* model using compounds that induce Ca^2+^ related cellular stress in HEK293T cells transfected with OCM or control plasmids further demonstrates the link between Ca^2+^ homeostasis and mitochondrial morphology. Thapsigargin (Tg) induces an increase in cytosolic Ca^2+^ by inhibiting Ca^2+^ uptake into the ER/SSC by binding to SERCA (120). Prolonged Tg administration induces mitochondrial fragmentation that leads to cell death (69, 121). Here, we show that prolonged Tg administration (48 h) induces mitochondrial fragmentation, as measured by decreased mitochondrial branching and size and reduced TOM20 labeling intensity. This mitochondrial fragmentation is rescued by expression of OCM-GFP. Similarly, administration of staurosporine, another inducer of apoptosis that increases cytosolic Ca^2+^ levels (122), leads to increased TUNEL staining in control (mCh-expressing) cells, but was rescued by the expression of OCM-mCh. As we previously reported (62), cultured cells transfected with OCM have reduced Ca^2+^ transients in response to both ATP and ionomycin. Although it is difficult to compare experiments where Ca^2+^ transients are induced rapidly in contrast to Tg administration over prolonged periods, both experiments are measuring, at least indirectly, OCM buffering capacity. Here, we have focused on the ability of OCM to buffer Ca^2+^ (although not directly measured) over a longer time period. Tg induces two phases of mitochondrial fragmentation; the first phase, which occurs in ~10 min, is reversible, while the second phase, which occurs after 32 h of Tg incubation, is irreversible, and induces apoptotic cell death (69). We believe that this second phase, which parallels the HEK293T Tg experiments reported here, applies more to sustained Ca^2+^ overload which may occur following traumatic noise exposure (123) or during aging. In OHCs, when Tg is administered, Ca^2+^ transients are prolonged, indicating a reduction in Ca^2+^ clearance (124). OCM, in addition to rapid Ca^2+^ buffering, may aid in Ca^2+^ clearance involved with prolonged cytosolic Ca^2+^ levels.

Thus, it may be reasonable to hypothesize that OCM protects cells against Ca^2+^-induced cell death by effectively maintaining Ca^2+^ homeostasis. Our *in vitro* cell culture model circumvents the challenges of *ex vivo* cochlear explants, which are extremely sensitive to culturing conditions and have a limited lifespan. Here, we use an *in vitro* cell culture model in conjunction with *in vitro* methods to corroborate our observations in OHCs in a more robust system.

In conclusion, both mobile Ca^2+^ buffers and mitochondria are likely to determine the ability of OHCs to compensate for large increases in Ca^2+^ after acoustic overexposure. Here, our results describe the cellular phenotypes associated with genetic deletion of *Ocm* and provide insight as to what makes *Ocm* KO OHCs more vulnerable to noise. Similar to other reports (24), our results suggest that the vulnerability of OHCs depends upon their ability to maintain Ca^2+^ homeostasis. These experiments, using a mouse model with a targeted deletion of *Ocm*, adds further support to the idea that Ca^2+^ dysregulation leads to an increased susceptibility to NIHL, and the induction of OHC death. Moreover, in *Ocm* KO OHCs, the observed changes in mitochondria, both in control and noise exposed mice, suggest that Ca^2+^ regulation may have a role in the overall integrity of mitochondria. We propose a mechanism whereby the lack of Ca^2+^ buffering by OCM predisposes *Ocm* KO OHCs to mitochondrial fragmentation and sensitizes OHCs to damage and loss (see Figure 8). Our findings provide further insight into the mechanisms mediating noise sensitivity and highlight the relationship between Ca^2+^ regulation and mitochondrial dynamics in OHCs.

## Data Availability

The raw data supporting the conclusions of this article will be made available by the authors, without undue reservation.

## Supplementary material

Supplementary Methods Visual schematic of how mitochondrial morphology was quantified in this study. **(A)** Shows how Imaris software was used in OHCs to calculate the Number of mitochondria/OHC volume (μm^3^) and Distance to Nearest Neighbor (μm) using Spot detection. We also show how Mitochondrial volume (μm^3^)/OHC volume (μm^3^), Sphericity, and Ellipticity (oblate and prolate) were calculated using the Surfaces feature. **(B)** Shows how mitochondrial branching was calculated for OHCs using the Skeletonize plugin in FIJI (70). **(C)** Shows how mitochondrial branching, size (μm^2^) and TOM20 intensity (integrated density) were calculated for HEK293T cell experiments using the Tubeness plugin in FIJI (71).

Supplementary Figure S1 Sphericity, distance to nearest neighbor, and ellipticity are not altered by deletion of Ocm or by noise administration. Apical and basal OHCs from mid-modiolar sections of unexposed and noise exposed Ocm WT and KO cochleae harvested 2 dpn. Mitochondrial morphology was quantified. **(A–D)** Bar graphs show mitochondrial **(A)** sphericity, **(B)** distance to nearest neighbor (μm), **(C)** ellipticity (oblate), and **(D)** ellipticity (prolate) in apical and basal OHCs of control and noise exposed Ocm WT and KO mice. *n* = 3–5 per group. All plotted values represent mean ± SEM. No statistical significance was found.

Supplementary Figure S2 Dose response of transfected HEK293T cells to STS administration. HEK293T cells were transfected with plasmid DNA encoding mCh (control) and administered varying doses of STS. Data was normalized as a percent of the mean of the untreated control (100%), where 0% was defined as the mean of the positive control for cell death (30% DMSO). Normalized data was fit using an inhibitor vs. normalized response (least squares fit) equation available in Prism. LD50 = 0.08290 μM STS (±0.017 SEM, *R*^2^ = 0.8682). Black dots represent means for each dose. *n* = 6 technical replicates for each dose. Bars represent SEM.
