# Supplementary material for: Absence of oncomodulin increases susceptibility to noise-induced outer hair cell death and alters mitochondrial morphology
Source: Front Neurol. 2024 Oct 23;15:1435749. doi: 10.3389/fneur.2024.1435749 (PMC11537894; doi:10.3389/fneur.2024.1435749)
Supplement: Supplementary Methods — Visual schematic of how mitochondrial morphology was quantified in this study. (A) Shows how Imaris software was used in OHCs to calculate the Number of mitochondria/OHC volume (μm3) and Distance to Nearest Neighbor (μm) using Spot detection. We also show how Mitochondrial volume (μm3)/OHC volume (μm3), Sphericity, and Ellipticity (oblate and prolate) were calculated using the Surfaces feature. (B) Shows how mitochondrial branching was calculated for OHCs using the Skeletonize plugin in FIJI (70). (C) Shows how mitochondrial branching, size (μm2) and TOM20 intensity (integrated density) were calculated for HEK293T cell experiments using the Tubeness plugin in FIJI (71). [file Data_Sheet_1.PDF]

Supplemental Methods

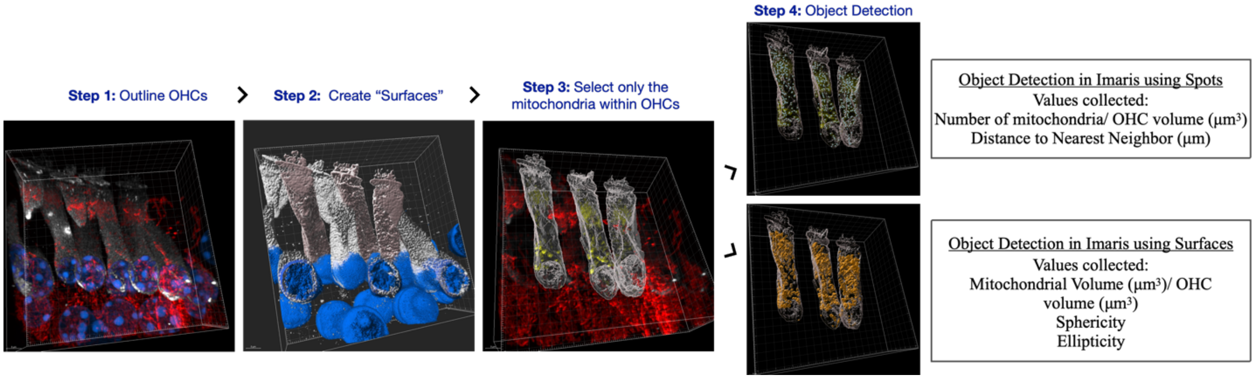

Supplemental Methods 1A. **Mitochondrial Morphology in OHCs using Imaris Software.** For mitochondrial morphology analysis of confocal z-stacks from mid-modiolar cochlear sections, object detection using the "Spots" feature (Number of mitochondria/OHC volume (μm^3) and Distance to Nearest Neighbor (μm)) and the "Surfaces" (Mitochondrial volume μm^3/ OHC volume (μm^3), Sphericity, and Ellipticity (oblate and prolate)) in Imaris (Oxford Instruments) was used.

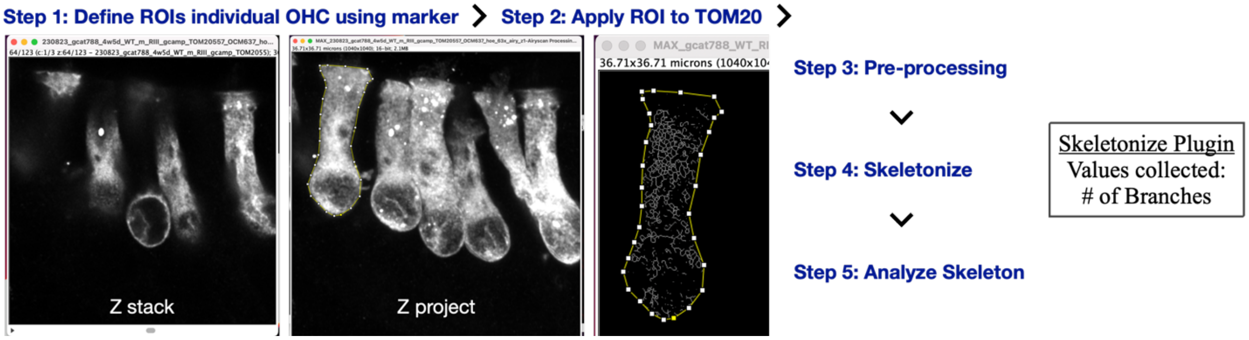

Supplemental Methods 1B. Mitochondrial Morphology in OHCs using FIJI. To calculate mitochondrial branching, the mean number of branches for individual OHCs was calculated using the ImageJ/FIJI Skeletonize plugin (Arganda-Carreras *et al.*, 2010). Due to the significant reduction in overall OHCs present in *Ocm* KO mice, particularly in basal regions, multiple z-stacks were taken in some cases to capture a minimum of 3 OHCs per region, per animal.

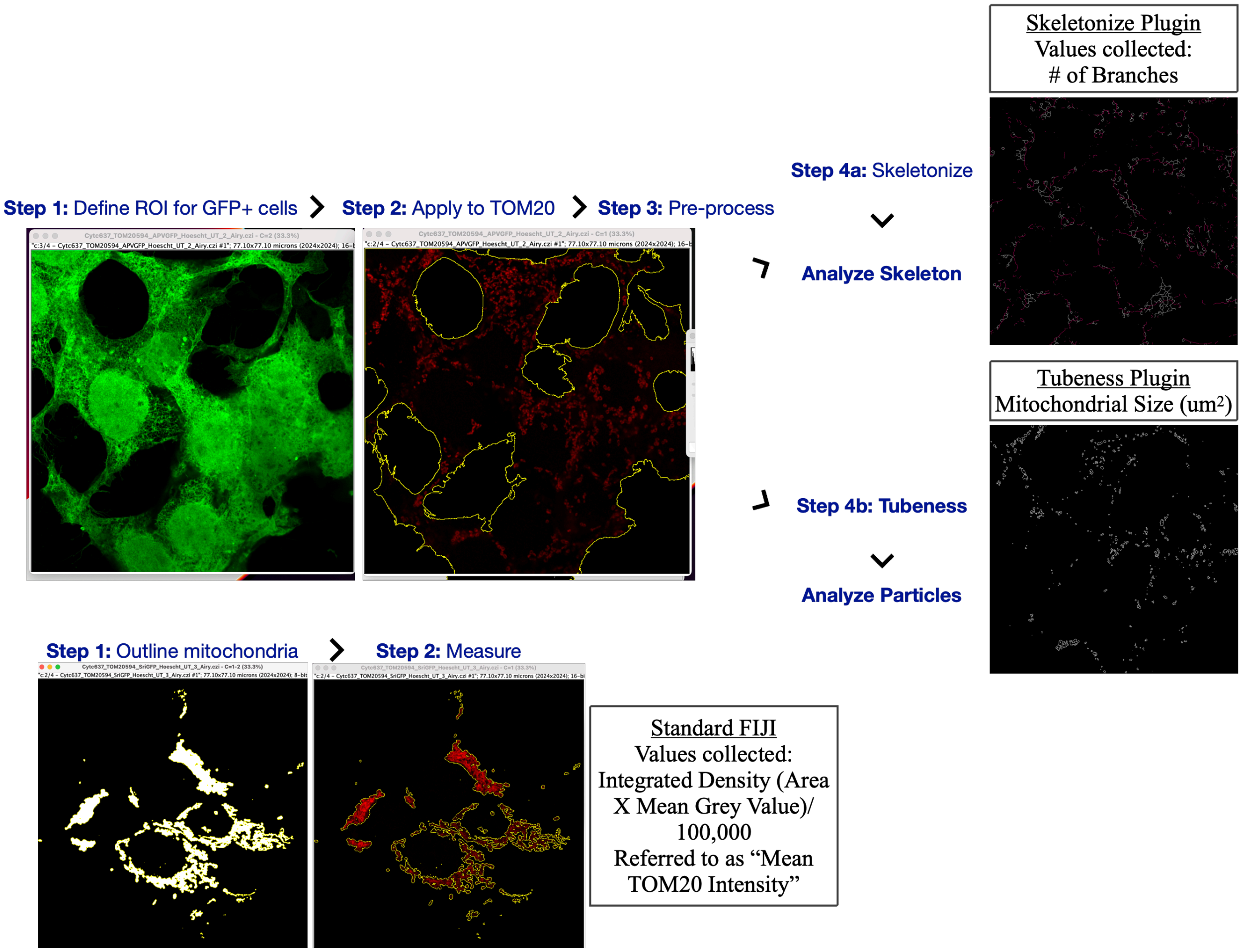

Supplemental Methods 1C. Mitochondrial Morphology in HEK293T cells using FIJI Software. For HEK293T mitochondrial branching, the Skeletonize plugin (Arganda-Carreras *et al.*, 2010) was applied to single confocal images. Only GFP+ HEK293T cells were analyzed. For mitochondrial size (μm^2), the ImageJ/FIJI "Tubeness" plugin was used (Sato *et al.*, 1998). Finally, mean TOM20 intensity for HEK293T cells was calculated by determining the Integrated Density (Area*Mean grey value) and divided by 100,000 for simplicity.
