# Supplementary figures and images for: Absence of oncomodulin increases susceptibility to noise-induced outer hair cell death and alters mitochondrial morphology
Source: Front Neurol. 2024 Oct 23;15:1435749. doi: 10.3389/fneur.2024.1435749 (PMC11537894; doi:10.3389/fneur.2024.1435749)

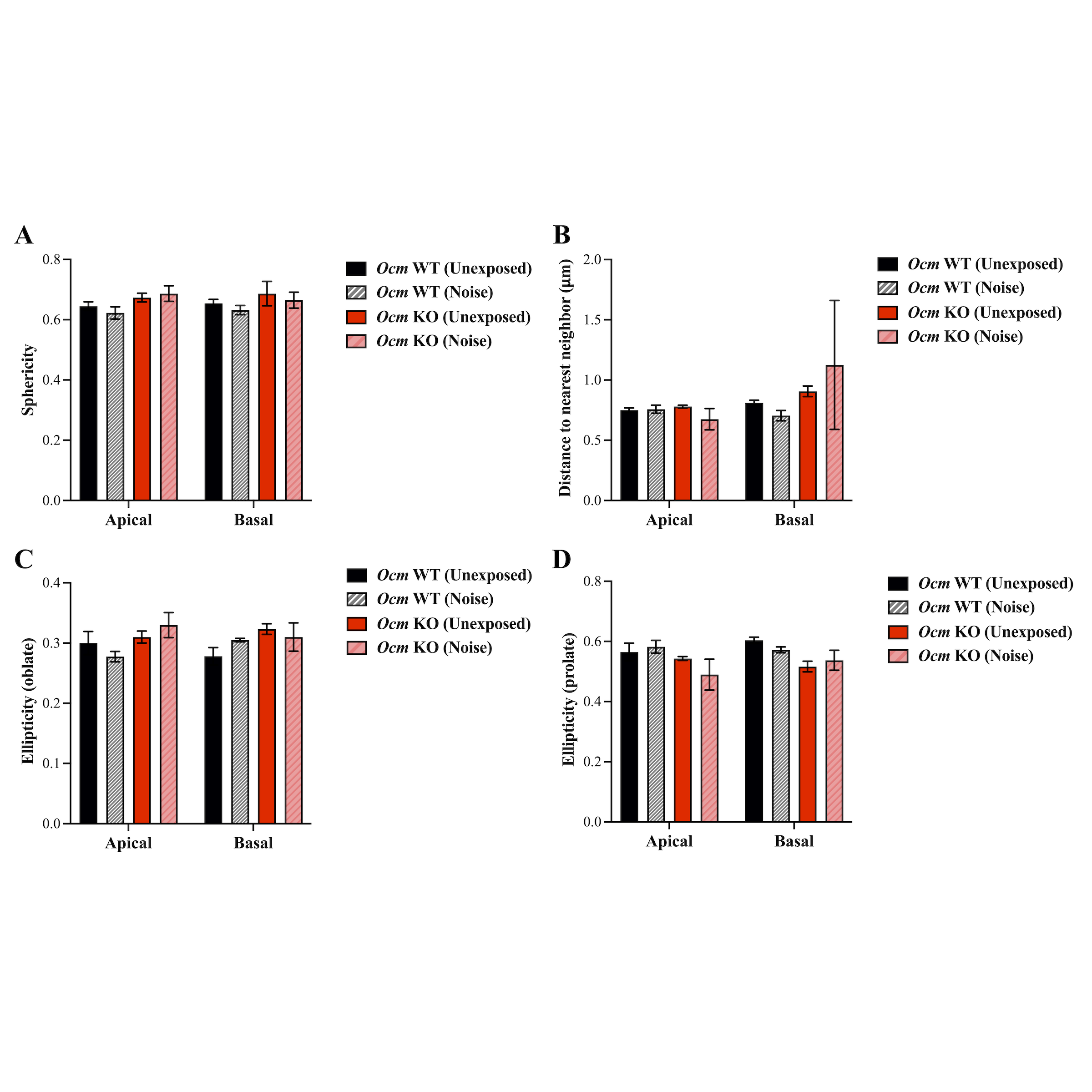

Supplement: Supplementary Figure S1 — Sphericity, distance to nearest neighbor, and ellipticity are not altered by deletion of Ocm or by noise administration. Apical and basal OHCs from mid-modiolar sections of unexposed and noise exposed Ocm WT and KO cochleae harvested 2 dpn. Mitochondrial morphology was quantified. (A–D) Bar graphs show mitochondrial (A) sphericity, (B) distance to nearest neighbor (μm), (C) ellipticity (oblate), and (D) ellipticity (prolate) in apical and basal OHCs of control and noise exposed Ocm WT and KO mice. n = 3–5 per group. All plotted values represent mean ± SEM. No statistical significance was found. [file Image_1.TIFF]

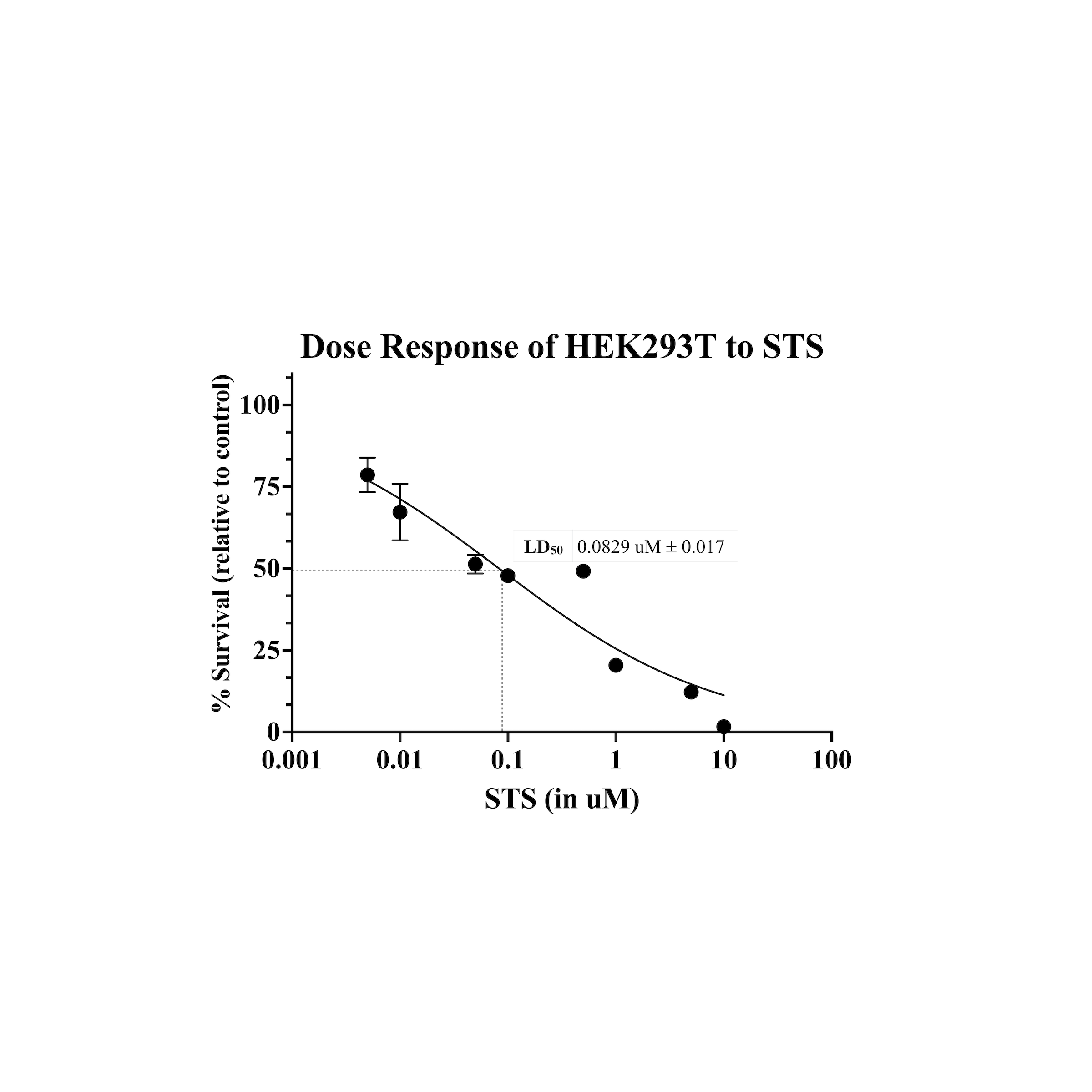

Supplement: Supplementary Figure S2 — Dose response of transfected HEK293T cells to STS administration. HEK293T cells were transfected with plasmid DNA encoding mCh (control) and administered varying doses of STS. Data was normalized as a percent of the mean of the untreated control (100%), where 0% was defined as the mean of the positive control for cell death (30% DMSO). Normalized data was fit using an inhibitor vs. normalized response (least squares fit) equation available in Prism. LD50 = 0.08290 μM STS (±0.017 SEM, R2 = 0.8682). Black dots represent means for each dose. n = 6 technical replicates for each dose. Bars represent SEM. [file Image_2.TIFF]
